# Synthesis of the Pentasaccharide Unit of the *Pseudomonas aeruginosa* Exopolysaccharide Psl Conjugation with CRM197, and Evaluation of Antigenicity in a QS-21/Pam_3_CSK_4_-Liposomal Formulation

**DOI:** 10.3390/molecules30081720

**Published:** 2025-04-11

**Authors:** Uzoamaka Clara Bokolo, Ravindika Dissanayake, Samir Ghosh, Shadia Nada, Babatunde S. Obadawo, Erin G. Prestwich, Katherine A. Wall, Steven J. Sucheck

**Affiliations:** 1 Department of Chemistry and Biochemistry, The University of Toledo, 2801 W. Bancroft Street, Toledo, OH 43606, USA; 2 Department of Medicinal and Biological Chemistry, The University of Toledo, 3000 Arlington Ave., Toledo, OH 43614, USA

**Keywords:** *Pseudomonas aeruginosa*, Psl exopolysaccharide, carbohydrate vaccine, β-mannoside, CRM197, QS21

## Abstract

Oligosaccharides and glycoconjugates play essential roles in various biological processes such as cellular recognition and signaling, and thus have attracted tremendous attention in the synthetic and biological communities over the past few decades. Contributing to this field, we have achieved the synthesis of the aminoxyglycoside pentasaccharide subunit of *Pseudomonas aeruginosa* polysaccharide synthesis locus (Psl) exopolysaccharide through an efficient 23 step process. This pentasaccharide was designed with an aminooxy derivative at the reducing end, which was used in a 2-step oxime-based bioconjugation to the protein carrier CRM197, with an epitope ratio of 1:4. The conjugate vaccine could generate anti-Psl antibodies that could recognize *P. aeruginosa* PAO1 bacteria and initiate opsonophagocytic killing of the bacteria. In addition, the aminoxyglycoside could be conveniently conjugated to a bifunctional aldehyde-biotin reagent, which can be used for quantifying antibody titers in vaccination studies.

## 1. Introduction

*Pseudomonas aeruginosa* (PA) belongs to the ESKAPE (*Enterococcus faecium*, *Staphylococcus aureus*, *Klebsiella pneumoniae*, *Acinetobacter baumannii*, *Pseudomonas aeruginosa*, and *Enterobacter* species) family of pathogens and was recognized as a widely distributed and concerning pathogen [1]. For example, carbapenem-resistant PA (CRPA) is now considered a serious public health threat, with some strains having limited treatment options [2]. PA, a Gram-negative bacterium, produces three exopolysaccharides: Psl, Pel, and alginate [3]. Mutational analysis has shown that Psl plays an important role in biofilm initiation in nonmucoid PA [4]. In addition, biofilm formation is stimulated when PA encounters Psl [5]. More recently, Psl has also been found to have important antimicrobial activity against *S. aureus* [6,7]. In support of the use of Psl as a vaccine antigen, broadly protective human antibodies to Psl have been known for years [8]. Further, it has been noted that 50% of mucoid clinical isolates from cystic fibrosis (CF) sputum were Psl-producing [9]. In addition, most PA isolates from keratitis cases were found to produce Psl and were susceptible to opsonophagocytic killing mediated by an anti-Psl monoclonal antibody [10]. More recent studies have shown that Psl-containing vaccines are protective in mice [8,11,12,13]. These reports suggest that Psl is a potential component for an anti-PA vaccine. CRM197, a non-toxic mutant form of the diphtheria toxin, was utilized in this study as a carrier protein conjugated to the Psl. This approach was taken because CRM197 has been extensively validated to significantly enhance the immunogenicity and protective efficacy of polysaccharide vaccines [14,15]. Polysaccharides alone are poorly immunogenic, especially in high-risk populations, such as infants and the elderly. They stimulate low-avidity antibody responses that lack immunological memory and rapid boosting ability. By chemically linking Psl to the highly immunogenic CRM197 protein, the immune system will mount a much more robust and durable anti-polysaccharide antibody response. The CRM197 carrier engages T cell help, converting the typical T-independent polysaccharide response to a T-dependent response. Thus, the switch results in higher titers of high-avidity IgG antibodies, affinity maturation, isotype switching, and the generation of long-lived plasma and memory B cells.

**Figure 1:**
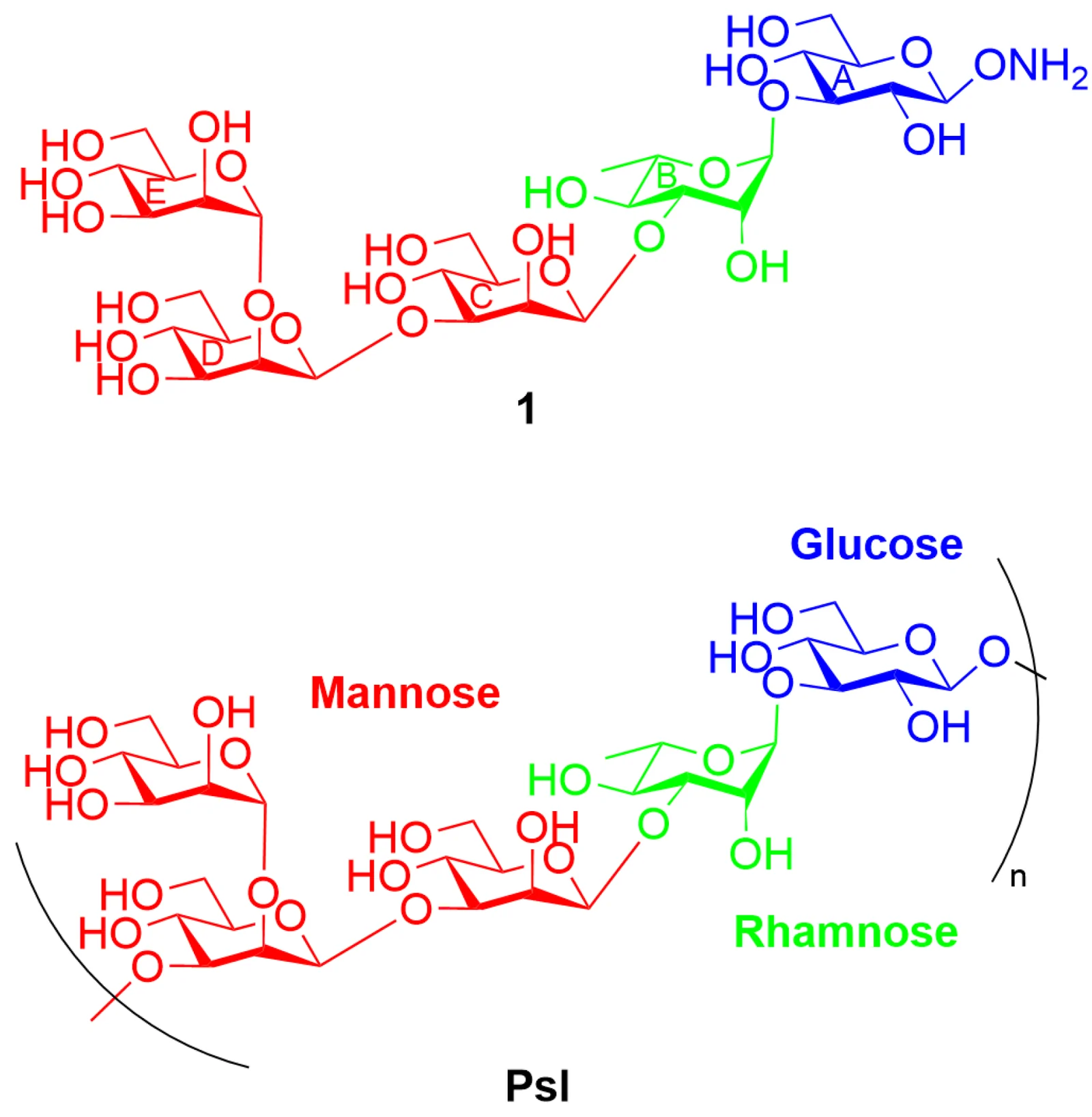
Structure of the target molecule **1**, and the repeating unit of the Psl exopolysaccharide of PA; n = number of repeating units.

Psl exopolysaccharide of PA is composed of neutral repeating pentameric subunits of D-mannose, L-rhamnose, and D-glucose residues connected through one α-and two β-mannosidic linkages (Figure 1) [9,12]. The previous syntheses of the Psl-repeating unit were reported by Li et al. in 2013 [12] and Demeter et al. 2020 [13]. The former study focused on the epitope mapping of monoclonal antibodies targeting the Psl exopolysaccharide and consisted of a stepwise synthesis in 34 steps. Demeter et al. later simplified the synthesis to 26 steps, utilizing a [1 + 3 + 1] block synthesis approach. Their work employed chemoselective preactivation-based mannosylation to simplify the synthesis of key building blocks, compared to the earlier 34-step approach by Li et al. In this study, we developed an efficient synthetic route to access the Psl pentasaccharide core, and the key innovation was the introduction of the aminooxy group, which expands the utility of this carbohydrate for further modifications and conjugations. In addition, we explored a step-saving [2 + 2 + 1] block synthesis approach.

**Figure 2:**
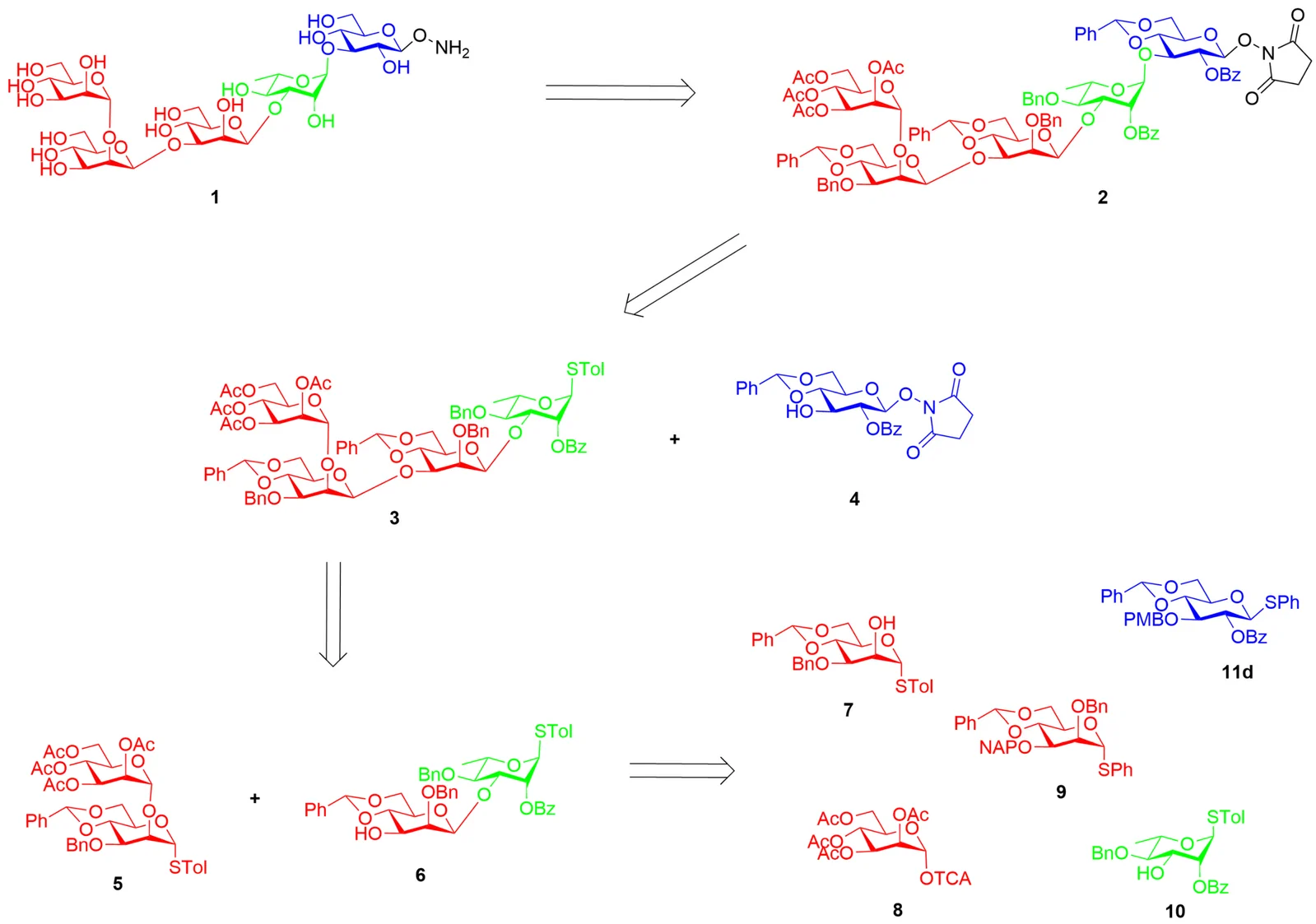
Retrosynthetic analysis of target compound. Color Scheme: Red = mannoside, Blue = glucoside, green = rhamnoside.

The Psl pentasaccharide target **1** consists of five units ABCDE (Figure 1), where ‘A’ is the reducing-end sugar and ‘E’ is the non-reducing-end sugar. The target contains two β-D-mannose units, ‘C and D’, one α-D-mannose unit, ‘E’, one α-L-rhamnose unit, ‘B’, and a β-D-glucose unit, ‘A’, in the form of an aminooxy glycoside (Figure 1). Psl pentasaccharide **1** was designed to be synthesized by exploiting a [2 + 2 +1] block glycosylation strategy (Figure 2). Our retrosynthetic analysis began with the target pentasaccharide **1**, which was obtained by deprotecting the protected pentasaccharide **2**. The protected pentasaccharide **2** is formed by glycosylating with glucose-based acceptor **4**. Tetrasaccharide **3** was synthesized by glycosylating acceptor **6** with donor **5** to form a 1,3-β mannoside linkage. Disaccharide **6**, containing a 1,3-β mannoside linkage, was synthesized first, followed by the synthesis of disaccharide **5** with an α mannoside linkage. The methods developed by Crich were employed to achieve the stereoselective formation of the β-mannoside linkages throughout the process [16].

## 2. Results and Discussion

**Scheme 1:**
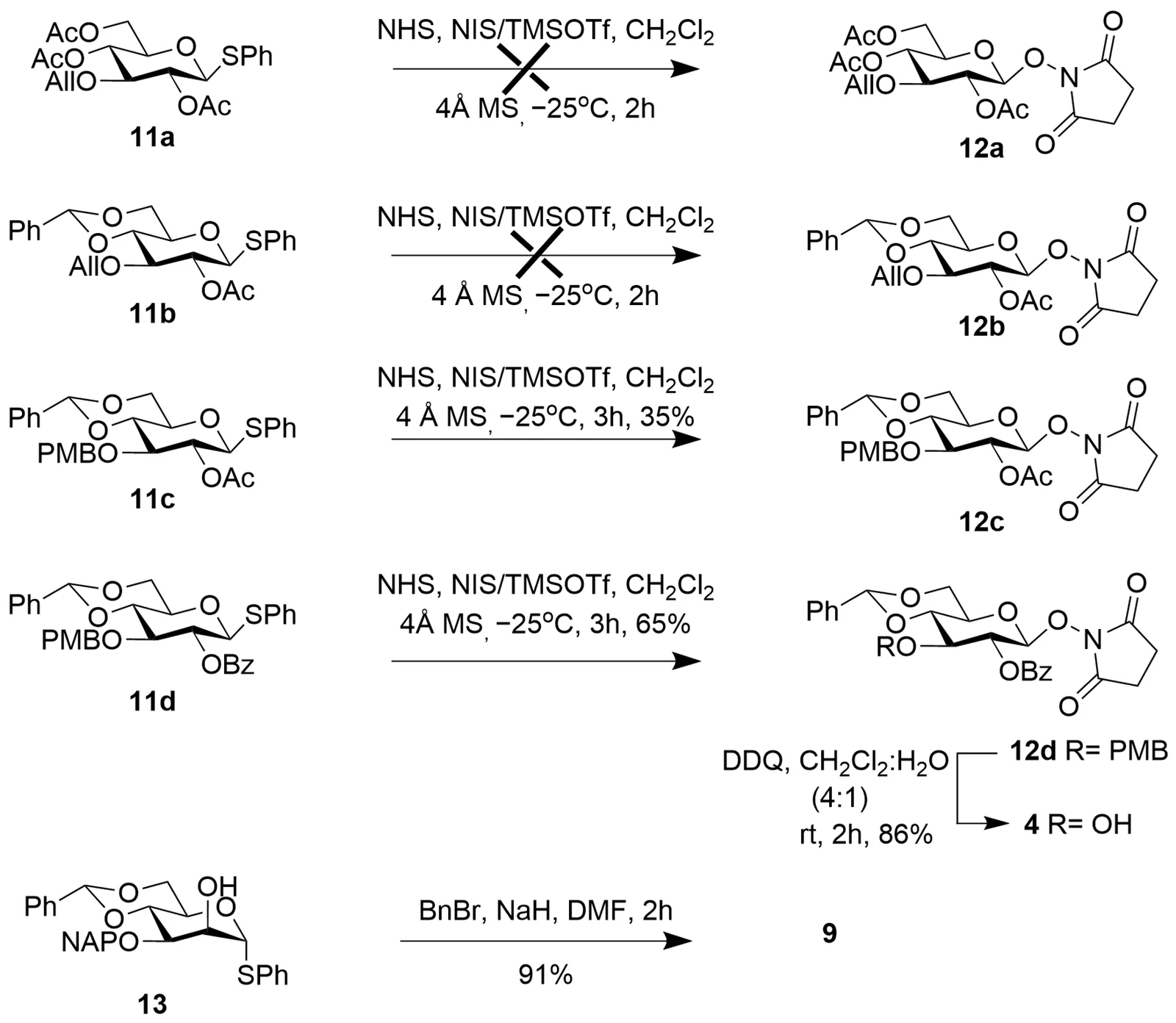
The monosaccharide building blocks **4** and **9**.

Suitably protected monosaccharides, **7** [17], **8** [18], **9** [17], **10** [19], and **11d** [20] (Figure 2) were prepared from the naturally available reducing sugars following literature methods. Compound **4**, Scheme 1, was prepared from compound **11d** [19,20] by stereoselective glycosylation with *N*-hydroxysuccinimide (NHS) in the presence of *N*-iodosuccinimide (NIS) and trimethylsilyl trifluoromethanesulfonate (TMSOTf) at 0 °C to furnish compound **12d** exclusively as the β-product in 65% yield [21]. The newly generated β-glycosidic bond in **12d** was confirmed by the large coupling constant (*J* = 6.25 Hz) of its anomeric ^1^H NMR signal at δ 5.31 ppm. We also went through trials with donors **11a**-**d** to prepare glycoside **12a**-**d**, which did not produce the desired targets or had low yields. Only donors **11c** and **11d** were viable with the active NHS acceptor using the NIS/TMSOTf promotor system to afford **12c** and **12d** 35% and 65% yields, respectively. Once compound **12d** was obtained, it was subjected to oxidative removal of the 4-methoxybenzyl (PMB) group using 2,3-dichloro-5,6-dicyano-1,4-benzoquinone (DDQ) to produce compound **4** in 84% yield [17,22]. D-Mannose building block **9** was obtained from compound **13** [23] by benzylation at the *O*-2 position using sodium hydride and benzyl bromide in 91% yield.

**Scheme 2:**
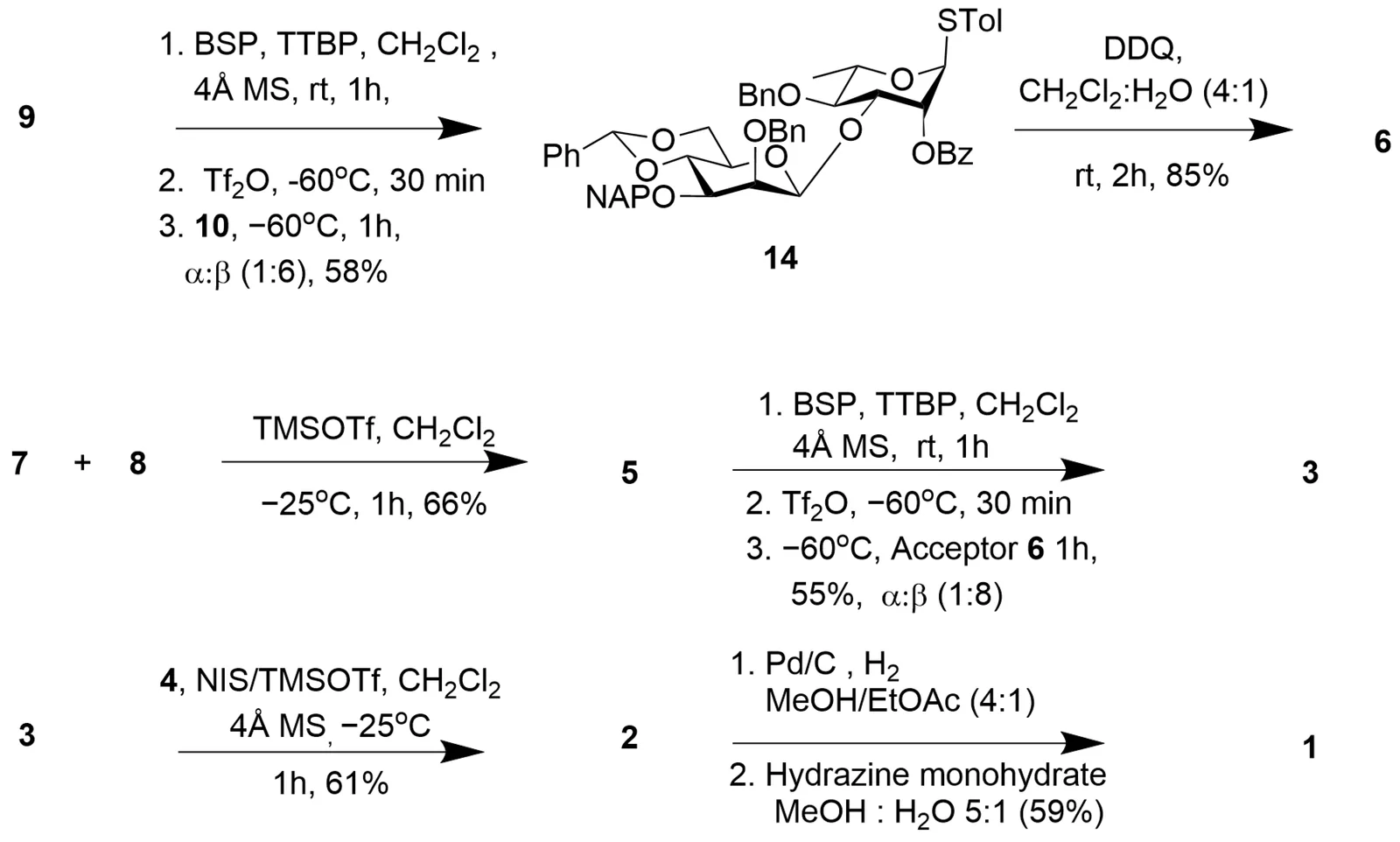
Synthesis of final pentasaccharide **1**.

Moving forward to the disaccharide **6** synthesis, we commenced with the formation of one of the two β-thioglycoside mannosidic linkages by employing the method developed by Crich. Thiomanoside **9** served as the donor and thiorhamnoside **10** as the acceptor, furnishing disaccharide **14** in 58% yield. The reaction resulted in a mixture of (1**→**3)-linked products in a 1:6 ratio of α-glycosylation, which were separated by flash column chromatography [23,24]. The formation of the β-mannosidic linkage was unambiguously established from the coupling constant (*J*_C1/H1_ = 158.8 Hz) value in the ^1^H-coupled ^13^C NMR spectrum of compound **14** [17,25]. Next, oxidative removal of the 3-*O*-(2-naphthyl)methyl (NAP) group of compound **14** using DDQ (2,3-dichloro-5,6-dicyano-1,4-benzoquinone) produced compound **6** in 85% yield (Scheme 2) [13].

To prepare tetrasaccharide **3**, we glycosylated D-mannose-derived thioglycoside **7** with mannosyl trichloroacetimidate **8** under Schmidt’s conditions to afford α disaccharide **5** in 66% yield. ^1^H NMR analysis showed signals at δ 5.15 (brS, 1 H, H−1_E_), 5.46 (d, *J* = 1.14 Hz, 1 H−1_D_), ^13^C NMR 99.7 (C-1E), and 88.3 (C-1D) [26]. The thiophenyl mannoside donor **5** was converted in situ into the corresponding anomeric triflate by treatment with 1-benzene-sulfinyl-piperidine (BSP), 2,4,6-tri-*tert*-butylpyrimidine (TTBP) and trifluoromethane sulfonic anhydride (Tf_2_O), at −60 °C, with the resulting triflate reacted immediately with the protected thiotollyl disaccharide acceptor **6**, in an S_N_2 fashion, to produce the expected β-mannosyl tetrasaccharide **3** α:β (1:8) in 55% [23].

### 2.1. Synthesis of the Aminooxy Pentasaccharide ***1***

With the key components available, we proceeded with the synthesis of pentasaccharide **1** by stereoselective glycosylation of donor **3** with acceptor **4** in the presence of the NIS-TMSOTf promoter system, which furnished pentasaccharide derivative **2** in 61% yield, together with a trace amount of the (1**→**3)-linked β-glycosylation product, which was separated by flash column chromatography [21]. Pentasaccharide **2** was subjected to hydrogenolysis using 10% Pd/C at 1 atm using a balloon filled with H_2_ to remove the benzylidene and benzyl groups [27]. After complete hydrogenolysis, as noted by TLC, the resulting residue was directly subjected to a Gabriel-type hydrazine hydrate reaction for the removal of the succinimide, giving rise to the aminooxy pentasaccharide **1** in 59% [28], which was purified by size exclusion chromatography on a P-2 gel, followed by lyophilization.

### 2.2. Synthesis of Psl Conjugates with CRM197, BSA, and Biotin

**Scheme 3:**
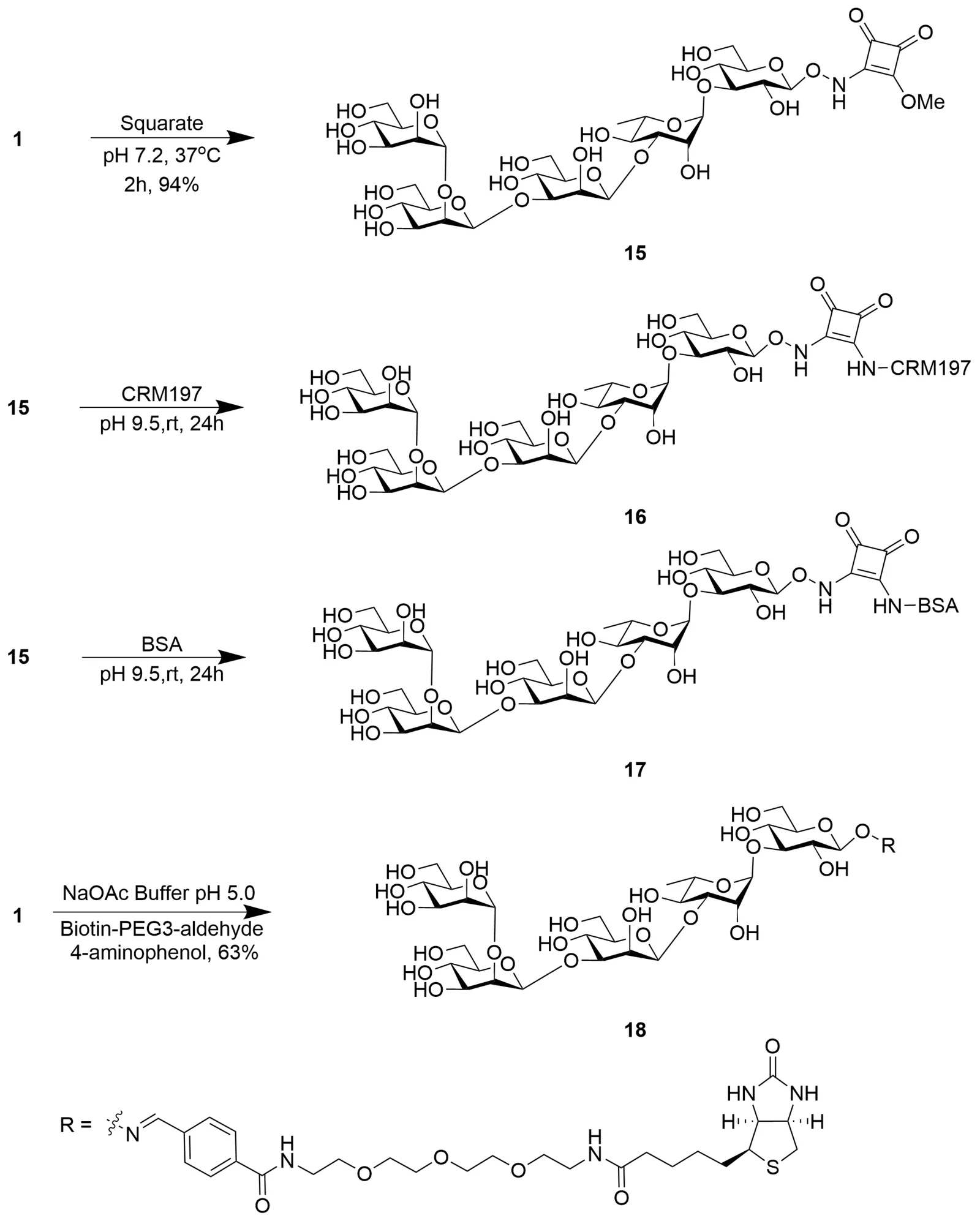
Psl conjugates **15–18**.

To conjugate pentasaccharide **1**, a squarate linker was utilized at pH 7.2 to produce compound **15**, which was purified by size exclusion chromatography on a bio gel P-2, followed by lyophilization to give compound **15** at 94% yield, and then conjugated to a carrier protein (CRM197) by incubation at pH 9.5 to form glycoconjugate **16** [29] (Scheme 3). The Psl epitope in **16** was measured using the phenol-sulfuric acid method using a standard curve for glucose, with absorbance readings taken at 480 nm [30]. The protein concentration was measured by the Bradford method using a Coomassie brilliant blue reagent as a standard curve for BSA as a reference, and the CRM197 carbohydrate protein epitope ratio was calculated to be 4.5. This result shows that about four to five copies of the carbohydrate were appended (Figure S15). The conjugation process utilized a squarate linker due to its ease of conjugation to amines under mild pH and temperature conditions, as well as the stability of the resulting amine. Glycoside **15** was also conjugated to bovine serum albumin (BSA) at pH 7.2 to give glycoconjugate **17**, and the epitope ratio was calculated to be 6, as shown in Figure S16 [29].

After successfully conjugating CRM197 to Psl polysaccharide, the bioconjugate was formulated into liposomes (DMPG/DMPC/Cholesterol/Pam_3_CysSK_4_/QS-21). This step allows for the potential encapsulation and delivery of the Psl-CRM197 conjugate using liposomal carriers, which are versatile nanocarriers widely employed in drug delivery applications. The liposomal formulation of the Psl-CRM197 conjugate using Pam_3_CysSK_4_:QS-21 as an adjuvant system could facilitate its targeted delivery, improve its stability, and modulate its release profile, among other advantages offered by liposomal drug delivery systems [31]. A biotin-conjugated version of compound **1** was synthesized **18** (Scheme 3) in 63%. This conjugate was utilized to measure and quantify the levels of anti-Psl antibodies in samples.

### 2.3. Vaccine Preparation Using Pam_3_CysSK_4_-QS-21 Liposomes and Psl-CRM197 Conjugate (***16***)

QS-21 was purified from Quil-A by RP-HPLC, as previously reported [32]. We found that a 30 mM formulation of lipids containing DMPG (4.4 mol%), DMPC (40.0 mol%), cholesterol (54.2 mol%), and Pam_3_CysSK_4_ (1.50 mol%), formulated with 1 µg QS-21 per 100 µL of injection solution, was well tolerated (Table 1) [32]. Thus, we prepared two sets of samples for vaccination. The DMPG/DMPC/Cholesterol/ Pam_3_CysSK_4_/QS-21 liposomes (labeled QS-21 liposomes) depicted in Table 2 are used as a control, while the DMPG/DMPC/Cholesterol/ Pam_3_CysSK_4_/QS-21/**16** liposomes (labeled Psl-CRM197 liposomes) illustrated in Table 2 contain the glycoconjugate. All liposomes were formulated using the extrusion method to a total final lipid concentration of 30 mM, sized using a 200 nm, followed by a 100 nm polycarbonate membrane. For the test vaccine (labeled Psl-CRM197 liposomes), conjugate **16** (500 μL of 0.56 mg/mL solution) was added to a 500 μL 2× stock solution of 60 mM liposomes to give a final lipid concentration of 30 mM that contained 0.28 mg/mL of **16**.

This solution was incubated at room temperature for 15 min, followed by vortexing at 180 rpm for 2 min. QS-21 (2.0 μL from 5.0 mg/mL stock solution of QS-21 in water) was added to this suspension, followed by vortexing the suspension at 180 rpm for 10 min immediately prior to injection. A summary of the formulations is shown in Table 2. The QS-21 + Psl-CRM197 (**16**) liposomes were also characterized by DLS. The QS-21 liposomes showed an R_h_ = 500.88 nm, ζ = 0.0057 ± 0.0014 V [32], while Psl-CRM197 liposomes showed an R_h_ = 1029.51 nm, ζ = 0.0016 ± 0.0014 V, respectively, as shown in Table S1 and Figure S19.

### 2.4. Vaccination Results

To assess the immunogenicity of the liposomal Psl-CRM197 vaccine, eight female C57BL/6J mice were randomly divided into two groups, each containing four mice. One group received 100 µL of the liposomal Psl-CRM197-QS-21vaccine, while the other group received 100 µL of the liposomal adjuvant. The mice were administered two booster doses at intervals of 14 days, and serum and spleen samples were collected at the conclusion of the vaccination schedule. The mice were bled before the immunization scheme and used as the control (referred to as pre-bleed).

**Figure 3:**
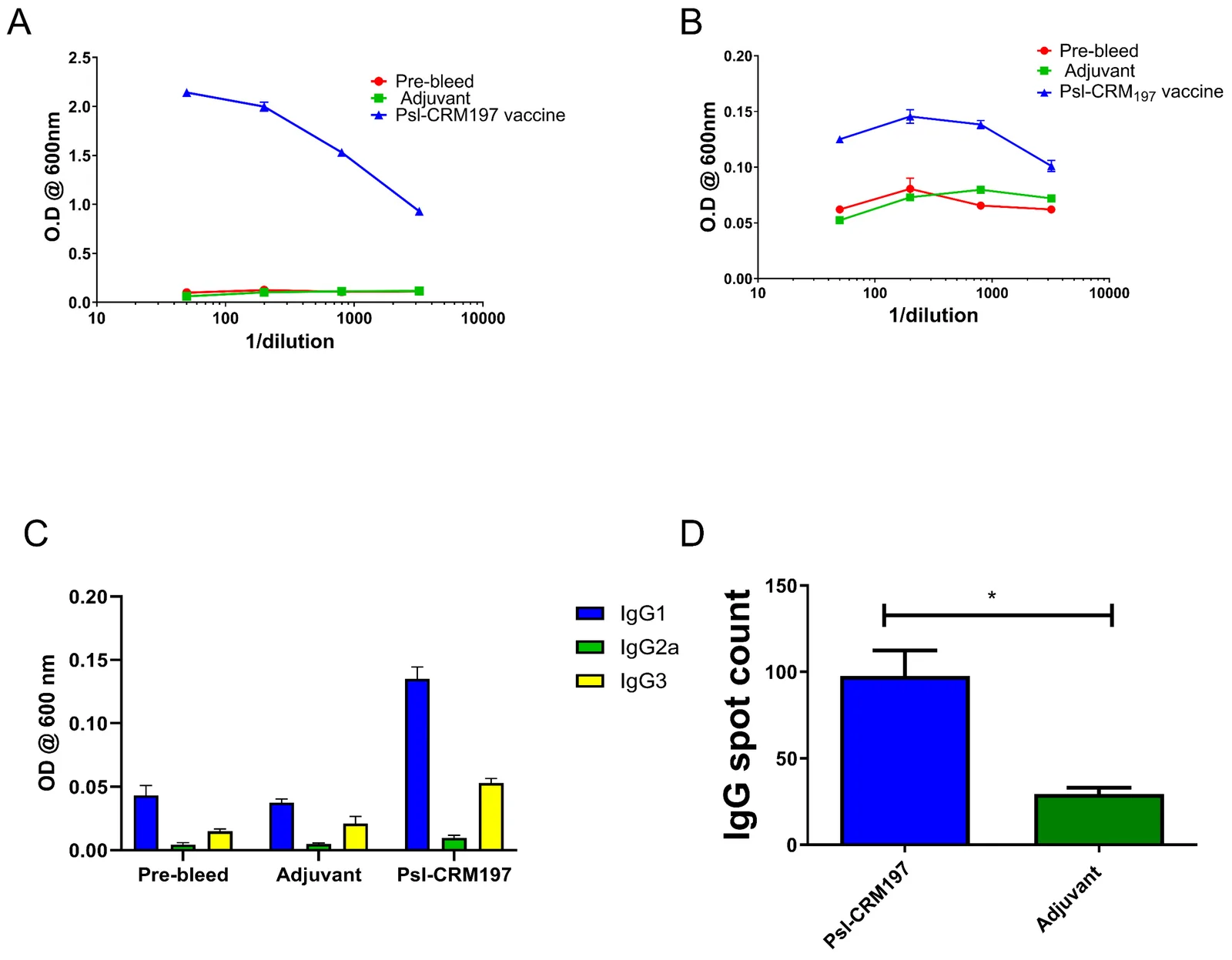
Anti-vaccine antibodies elicited by vaccination. ELISA of sera using plates coated with (**A**) Psl-CRM197 or (**B**) Psl-biotin and streptavidin. Secondary antibody was anti-mouse IgG (IgG specific). (**C**) IgG isotype-specific ELISA on plate coated with Psl-biotin and streptavidin. Mouse serum was used at 1:200 dilution. Horseradish peroxidase (HRP) conjugated goat anti-mouse IgG1, IgG2a, and IgG3 (1:5000) were used as secondary antibodies. (**D**) ELISPOT assay for IgG-producing B cells using a Mouse IgG Single-Color ELISPOT kit by ImmunoSpot^®^. * = *p* < 0.05.

Figure 3 shows the antibody titers of the two groups against Psl-CRM197 Figure 3A and Psl-biotin Figure 3B. The isotype-specific ELISA outcomes presented in Figure 3C illustrate the presence of IgG1, IgG2a, and IgG3 antibodies, indicating a mixed Th1/Th2 environment. The IgG ELISPOT result in Figure 3D further confirms the presence of significant amounts of IgG producing B cells in the Psl-CRM197 vaccine group.

**Figure 4:**
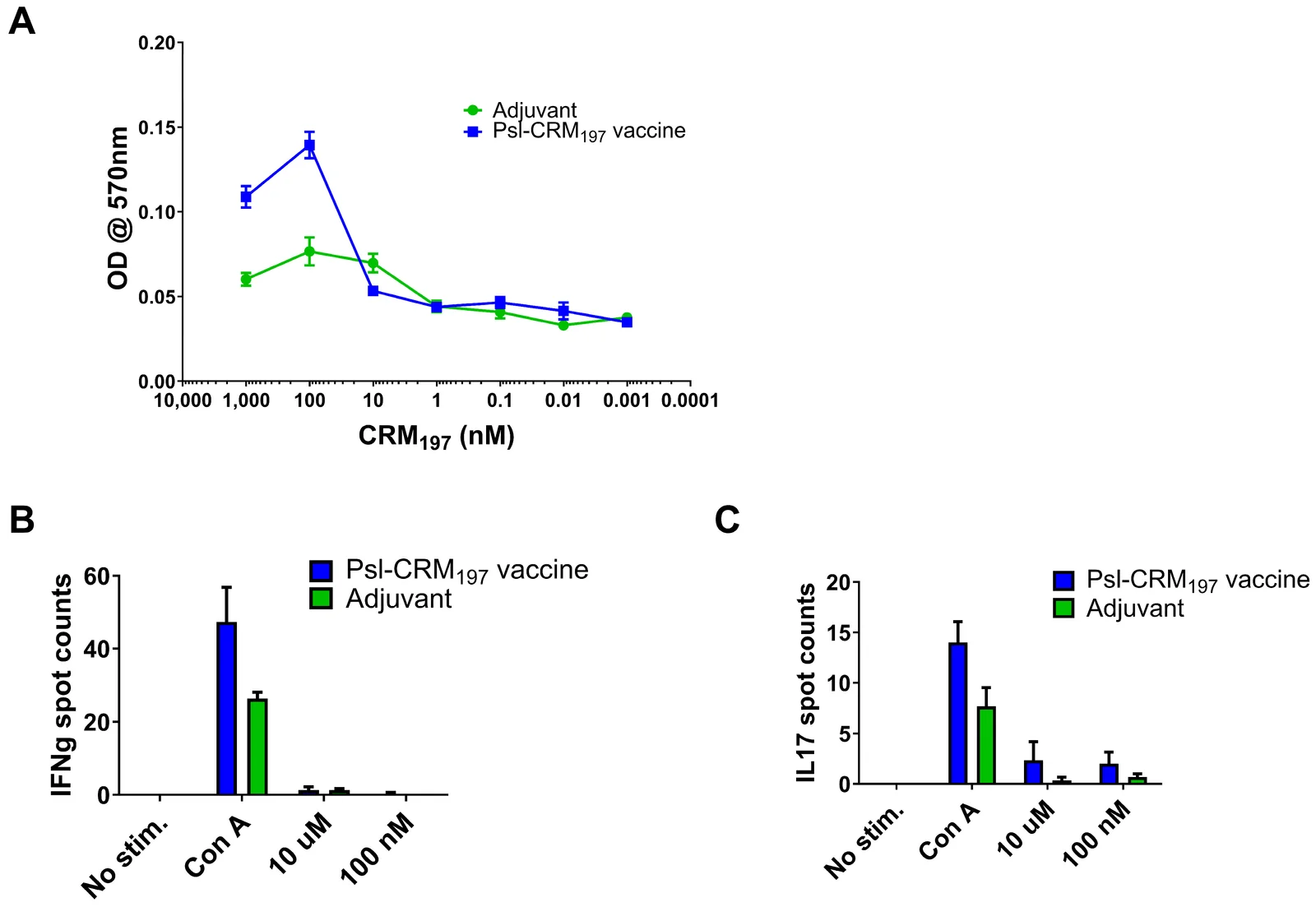
(**A**) T cell proliferation assay. Spleen cells harvested at day 42 were incubated with varying concentrations of Psl-CRM197 antigen for 6 days and developed using MTT reagent. ELISPOT assay for (**B**) IFN-γ- or (**C**) IL-17-producing T cells using the Double-Color Murine IFN-γ/IL-17 ELISPOT kit by ImmunoSpot^®^. Spleen cells were harvested on day 36 and incubated with different concentrations of Psl-CRM197 antigen. Concanavalin A at 2 µM was used as a positive control.

Spleen cells obtained from the vaccinated mice were subjected to stimulation with the Psl-CRM197 antigen across a spectrum of concentrations spanning from 1000 nM to 0.001 nM. The stimulated cells were assessed using the 3-[4,5-dimethylthiazol-2-yl]-2,5 diphenyltetrazolium bromide (MTT) reagent to detect the degree of T cell proliferation. As illustrated in the accompanying Figure 4A, the vaccine group exhibited greater T cell proliferation at higher concentrations compared to the control group. IFN-γ/IL-17 double-color ELISPOT results in Figure 4B,C indicate IFN-γ- and IL-17-producing T cells in the vaccinated mice. Accordingly, the Psl-CRM197-adjuvant group shows the presence of both IFN-γ and IL-17 when higher concentrations of a Psl-CRM197 antigen were used to stimulate the cells.

**Figure 5:**
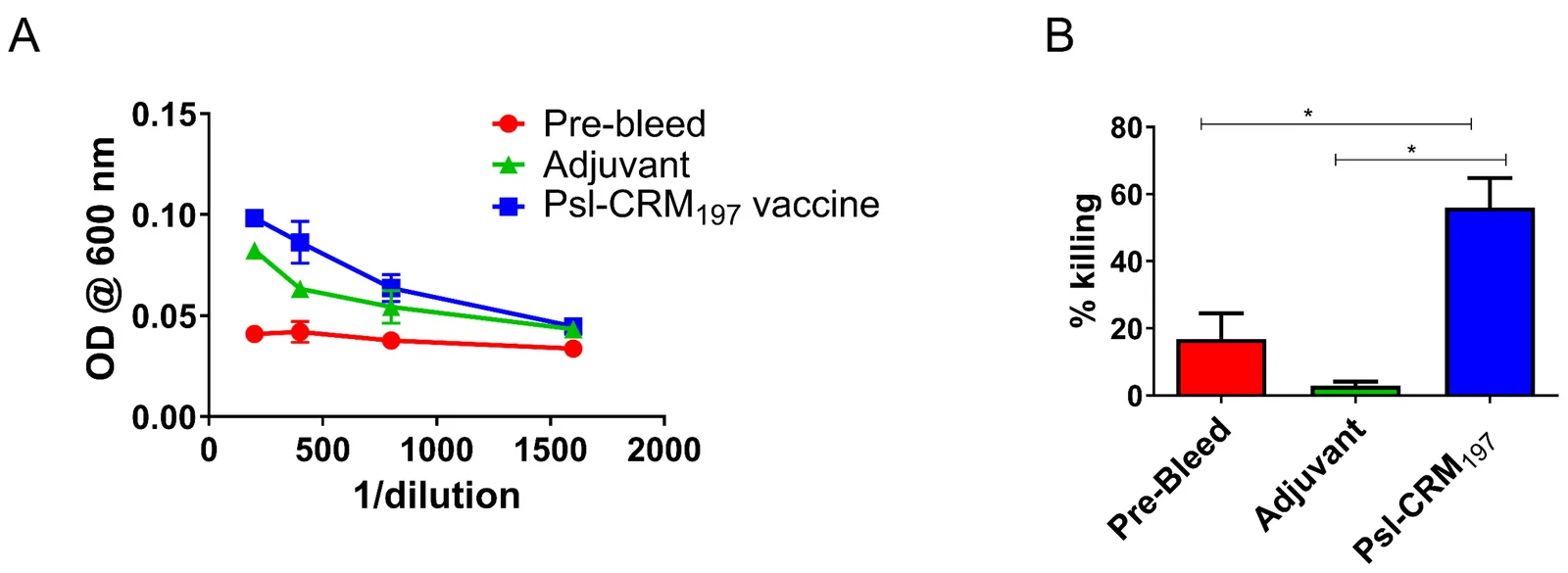
(**A**) Whole cell ELISA assay of the pooled sera on a plate coated with intact *P. aeruginosa* (PAO1 strain) bacteria. HRP-conjugated goat anti-mouse IgG-specific antibody was used as the secondary antibody. (**B**) Opsonophagocytic killing assay. Log-phase *P. aeruginosa* (PAO1 strain) were incubated with pooled sera, RAW264.7, and rabbit complement and plated on LB agar plates to determine the survival percentage of bacteria. * = *p* < 0.05.

Based on the findings from the whole cell ELISA analysis (Figure 5A), it was determined that the antibodies produced by the vaccine can effectively identify intact PAO1 PA bacteria. Notably, a significant reduction in bacterial count was observed when these bacteria were incubated with mouse serum, rabbit complement, and mouse macrophage RAW264.7 cells. The killing percentage for the Psl-CRM197 vaccine group was 56% (Figure 5B). This indicates that the vaccine can recognize and kill intact bacteria and may therefore be able to protect the host during an actual infection.

Anti-Psl antibodies provide some protective functions not produced by a protein-only vaccine. Psl is involved in helping PA attach to target cells; therefore, antibodies may reduce infectivity. The distribution of anti-carbohydrate isotypes produced by this vaccine broadens their functional activity, as IgG3 antibodies provide strong complement-activating and opsonophagocytic activity. Inhibition by non-complement activating antibodies has been observed in patients with severe respiratory infections [33], thus the desire to shift away from a solely TH2 response. Protection in this vaccine model is due to anti-carbohydrate antibodies and cytokines produced during the vaccination response. In the future, we plan to compare the Psl-CRM197 vaccine to a Psl-OprF vaccine that, in addition, promotes the generation of anti-PA memory T cells that can be stimulated to produce cytokines during a PA challenge.

## 3. Experimental Section

### 3.1. General Methods

All chemicals and solvents were analytical reagent-grade chemicals obtained from Acros Organics, Fischer Scientific, Alfa Aesar (all Thermo Fisher Scientific, Waltham, MA, USA), and Sigma-Aldrich (St. Louis, MO, USA). Silica gel (230–400 mesh) for flash column chromatography was obtained from Sorbent Technologies, Inc. (Norcross, GA, USA). The dichloromethane used in glycosylation reactions was dried using activated 4 Å molecular sieves. Reactions were performed in flame-dried glassware, monitored using thin-layer chromatography (silica gel 60, f254), and the spots were visualized by UV light or by charring with 5% H_2_SO_4_. The ^1^H NMR and ^13^C NMR spectra of the compounds were recorded utilizing residual CHCl_3_ or D_2_O as internal references on an Avance III 600 MHz spectrometer (Bruker, Billerica, MA, USA). High-resolution mass spectrometry (HRMS) measurements of the new compounds were conducted at the Department of Chemistry and Biochemistry, University of Toledo, using a TM Synapt High-Definition Mass Spectrometer (Waters, Milford, MA, USA), performed on a Micromass Q-TOF 2 (Waters, Milford, MA, USA) instrument. Low-resolution mass spectrometry (LRMS) measurements of the new compounds were conducted at the Department of Chemistry and Biochemistry, University of Toledo, using a Finnigan LCQ Deca Mass Spectrometer instrument (Thermo Fisher Scientific, Waltham, MA, USA). A Litesizer 500 dynamic light scattering (DLS) instrument (Anton Paar, Graz, Austria) with the refractive index set to auto was employed to analyze particle size and ζ potential. Polystyrene cuvettes (10 mm × 10 mm × 45 mm) and polycarbonate Omega cuvettes with male Luer plugs (350 μL), obtained from Anton Paar (Graz, Austria), were utilized to determine particle size and ζ potential, respectively. Deionized water was generated from a GenPure Pro UV instrument (Thermo Fisher Scientific, Waltham, MA, USA) and was used for all media and buffer preparations, while ultrapure distilled Invitrogen DNase- and RNase-free water (Thermo Fisher Scientific, Waltham, MA USA) was employed for vaccine preparation. An Eppendorf 5810R centrifuge (Hamburg, Germany) was used for microcentrifugation. The final vaccine formulation utilized 1× phosphate-buffered saline (PBS) buffer pH = 7.4 (endotoxin-free) purchased from Thermo Fisher Scientific (Waltham, MA, USA). SDS-PAGE was performed using a Mini Gel Tank (Thermo Fisher Scientific, Waltham, MA, USA) fitted with Life Technologies Novex™ Tricine Mini Protein Gels, 16% (Thermo Fisher Scientific, Waltham, MA USA), powered by a PowerEase-90W power supply (Thermo Fisher Scientific, Waltham, MA, USA). The gels were run at 120 V for 2 h, and a prestained Invitrogen molecular-weight ladder (10–250 kDa) (Thermo Fisher Scientific, Waltham, MA, USA) served as a protein standard. After electrophoresis, the gels were stained by adding a staining solution (Brilliant Blue R dye: 1 g/L, methanol: 45%, acetic acid: 10%, H_2_O: 45%) and shaking on a Rotator Orbital Shaker (Vevor, Shanghai, China) at 80 rpm at room temperature for 10 min. Subsequently, the gels were destained by soaking in a destaining solution (methanol: 45%, acetic acid: 10%, H_2_O: 45%) and shaking at 75 rpm on a Vevor Rotator Orbital Shaker until completely destained, with Kimtech Science Kimwipes® Low-Lint Wipers (Kimberly-Clark Professional, Roswell, GA, USA) used to absorb the dye. Matrix-assisted laser desorption/ionization (MALDI)-TOF analysis was performed on a Bruker Micro-flex (Billerica, MA, USA) bench-top instrument calibrated with BSA and run in linear mode. The matrix used for MALDI analysis was Super-DHB which contains 2,5-dihydroxybenzoic acid:2-hydroxy-5-methoxybenzoic acid (9:1) () (MilliporeSigma, Burlington, MA, USA) at a concentration of 40 mg/mL. Purification of QS-2l from Quil-A® adjuvant (InvivoGen, San Diego, CA, USA) was carried out using a LC-20AP preparative HPLC pump (Shimadzu, Kyoto, Japan) connected to a Shimadzu FRC10A fraction collector, equipped with an Ultra C8 5 μm, 150 × 10.0 mm^2^ column (Restek, Centre County, PA, USA). Liposome sizing was performed using a LiposoFast-Basic extruder (AVESTIN, Inc., Ottawa, ON, Canada).

### 3.2. O-Succinimidyl-3-O-(4-Methoxybenzyl)-4,6-O-Benzylidene-2-Benzoyl-β-D-Glucopyranoside (***12d***)

To a solution of compound **11d** [19] (115 mg, 0.19 mmol) and *N*-hydroxy succinimide (NHS) [28] (44 mg, 0.38 mmol) in anhydrous CH_2_Cl_2_ (5 mL) were added to preactivated 4 Å molecular sieves. The reaction mixture was stirred under nitrogen at room temperature for 45 min. The reaction mixture was cooled to −30 °C, and *N*-iodosuccinimide (56 mg, 0.25 mmol) was added. After 10 min, trifluoromethanesulfonate (TMSOTf; 10 μL) [34] was added, and the reaction mixture was stirred until completion of the reaction (1 h), as noted by TLC. The reaction mixture was quenched with saturated aqueous NaHCO_3_ and aqueous 5% Na_2_S_2_O_3_, filtered through a bed of Celite^®^-545, and the bed was washed with CH_2_Cl_2_. The organic layer was washed 3 times with saturated aqueous NaHCO_3_ (20 mL) and water (40 mL), dried over anhydrous Na_2_SO_4_, and concentrated under reduced pressure to give a crude product. This crude material was purified by flash column chromatography on silica gel using toluene/EtOAc (2:1) as the eluent to furnish pure β-compound **12d** (76 mg, 65%). *R_f_* = 0.32 (2:1 toluene/EtOAc); ^1^H NMR (600 MHz, CDCl_3_) δ 8.08–6.68 (m, 14 H, Ar-H), 5.68 (s, 1 H, PhC*H*), 5.56 (t, *J* = 6.36 Hz, 1 H, H-2), 5.31 (d, *J* = 6.25 Hz, 1 H, H-1), 4.79 (d, *J* = 11.88 Hz, 1 H, PhC*H*_2_), 4.72 (d, *J* = 11.88 Hz, 1 H, PhC*H*_2_), 4.35–4.33 (m, 2 H, H-4, H-6), 3.98 (t, *J* = 10.32 Hz, 1 H, H-6), 3.91–3.90 (m, 1 H, H-3), 3.88 (OC*H*_3_), 3.68–3.67 (m, 1 H, H-5), 2.70 (br, 4H, succinimide). ^13^C NMR (125 MHz, CDCl_3_) δ 170.38 (2 C, -C(O)CH_2_-CH_2_-C(O)), 165.03 (COPh), 159.2–113.63 (Ar-C), 103.58 (C-1), 101.23 (Ph*C*H), 79.81 (C-4), 77.32 (C-3), 73.14 (OBn), 71.49 (C-2), 68.3 (C-6), 66.42 (C-5), 55.15 (O*C*H_3_), 25.4 (succinimide). ESI-HRMS [(M + H)^+^] calcd for C_32_H_32_NO_10_: 590.2026; found: 590.2030.

### 3.3. O-Succinimidyl-4,6-O-Benzylidene-2-Benzoyl-β-D-Glucopyranoside (***4***)

To a solution of compound **12d** (0.4 g, 0.68 mmol) in CH_2_Cl_2_ (5 mL) was added an emulsion of DDQ [22] (0.23 g, 1.02 mmol) in H_2_O (20 mL). The reaction mixture was stirred at room temperature for 2 h. The reaction mixture was diluted with CH_2_Cl_2_ (30 mL), and the organic layer was washed with saturated aqueous NaHCO_3_ (30 mL) and water (30 mL), dried over anhydrous sodium sulfate (Na_2_SO_4_), and concentrated. The crude product was purified by silica gel chromatography (3:1toluene/EtOAc) to give **4** (264 mg, 84%) as a solid. *R_f_* = 0.25 (3:1 toluene/EtOAc); ^1^H NMR (600 MHz, CDCl_3_) δ 8.18–7.20 (m, 10 H, Ar-H), 5.61 (s, 1 H, PhC*H*), 5.44 (t, *J* = 7.08 Hz, 1 H, H-2), 5.35 (d, *J* = 6.90 Hz, 1 H, H-1), 4.37–4.34 (m, 1 H, H-6), 4.13–4.11 (m, 1 H, H-3), 4.04 (t, *J* = 9.36 Hz, 1 H, H-4), 3.94 (t, *J* = 10.26 Hz, 1 H, H-6), 3.64–3.62 (m, 1 H, H-5), 2.70 (br, 4H, succinimide). ^13^C NMR (125 MHz, CDCl_3_) δ 170.4 (2 C, -C(O)CH_2_-CH_2_-C(O)), 166.0 (COPh), 137.9–125.32 (Ar-C), 103.6 (C-1), 101.9 (Ph*C*H), 79.7 (C-4), 73.3 (C-2), 72.5 (C-3), 68.2 (C-6), 66.5 (C-5), 25.4 (succinimide). ESI-HRMS [(M + H)^+^] calcd for C_24_H_24_NO_9_: 470.14510; found: 470.1450.

### 3.4. Tolyl-2-O-Benzyl-4,6-O-Benzylidene-3-O-Napthyl-1-Thio-α-D-Mannoyranoside (***9***)

To a solution of compound **13** [35] (3.6 g, 7.2 mmol) in anhydrous DMF (30 mL), NaH (60% in mineral oil, 1.5 eq) was added in portions under an argon atmosphere at 0 °C. After the evolution of the hydrogen ceased, benzyl bromide (1.2 eq) was added dropwise, and the mixture was stirred at 25 °C for 3 h. After completion, the reaction mixture was quenched by the addition of ice-cold water (100 mL), and a solid precipitate was obtained. A solid was obtained by filtration. The solid was dissolved in EtOAc (100 mL) and washed with brine (100 mL), dried over anhydrous sodium sulfate (Na_2_SO_4_), and concentrated in vacuo. The crude residue was purified by silica gel column chromatography to afford compound **9** (4.0 g, 91%) as a colorless viscous oil. *R_f_* = 0.58 (7:3 *n*-hexane/EtOAc); ^1^H NMR (600 MHz, CDCl_3_) δ 7.95–7.36 (m, 22 H, Ar-H), 5.80 (s, 1 H, PhC*H*), 5.67 (d, *J* = 0.12 Hz, 1 H, H-1), 5.07 (d, *J* = 12.48 Hz, 1 H, PhC*H*_2_), 4.94 (d, *J* = 12.48 Hz, 1 H, PhC*H*_2_), 4.49–4.84 (m, 2 H, PhC*H*_2_), 4.51 (t, *J* = 9.54 Hz, 1 H, H-4), 4.47–4.43 (m, 1 H, H-5), 4.38–4.36 (m, 1 H, H-6), 4.22–4.21 (m, 1 H, H-2), 4.19–4.17 (m, 1 H, H-3), 4.03 (t, *J* = 10.14 Hz, 1 H, H-6). ^13^C NMR (125 MHz, CDCl_3_) δ137.8–125.8 (Ar-C), 101.7 (PhCH), 87.2 (C-1), 79.2 (C-4), 78.1 (C-2), 76.4 (C-3), 73.2 (ArCH_2_), 73.0 (OBn), 68.7 (C-6), 65.7 (C-5). ESI-HRMS [(M + Na)^+^] calcd for C_37_H_34_NaO_5_S: 613.2024; found: 613.2050.

### 3.5. Tolyl-[2-O-Benzyl-4,6-O-Benzylidene-3-O-Napthyl-β-D-Mannopyranosyl]-(1→3)-2-O-Benzoyl-4-O-Benzyl-1-Thio-α-L-Rhamnopyranoside (***14***)

A mixture of thiomannosyl donor **9** (100 mg, 0.17 mmol), 1-(phenylsulfinyl)piperidine (BSP) (49.6 mg, 0.23 mmol, 1.4 equiv), 2,4,6-tri-*tert*-butylpyrimidine (TTBP) [24,36,37] (84 mg, 0.33 mmol, 2 equiv), and 4 Å molecular sieves in CH_2_Cl_2_ (3 mL) was stirred under an atmosphere of argon for 1 h. The reaction was cooled to −60 °C and trifluoromethane sulfonic anhydride (Tf_2_O) (57 µL, 0.20 mmol, 1.2 equiv) was added. After 30 min of stirring at −60 °C, a solution of the acceptor **10** [38] (118 mg, 0.25 mmol, 1.5 equiv) in CH_2_Cl_2_ (1 mL) was added using a cannula wire. The reaction mixture was stirred for a further 1 h at −60 °C and then quenched by the addition of triethyl phosphite (42 µL, 0.25 mmol). The mixture was filtered, and the filtrate was washed with saturated aqueous solution of NaHCO_3_ (40 mL) and brine (40 mL). The organic phase was dried with anhydrous Na_2_SO_4_, filtered, and the filtrate was concentrated under reduced pressure. The crude product was purified by silica gel flash column chromatography to give **14** (95 mg, 59%) as a colorless liquid. *R_f_* = 0.49 (3:1 *n*-hexane/EtOAc); ^1^H NMR (600 MHz, CDCl_3_) δ 8.25–7.15 (m, 31 H, Ar-H), 5.66–5.65 (m, 1 H, 2_B_), 5.62 (s, 1 H, PhC*H*), 5.59 (brS, 1 H, 1_B_), 4.82–4.68 (m, 6 H, 1_C_, 5 PhC*H*_2_), 4.60 (d, *J* = 11.34 Hz, 1 H, PhC*H*_2_), 4.37–4.32 (m, 2 H, 6_C_, 5_B_), 4.25–4.23 (m, 1 H, 3_B_), 4.19 (t, *J* = 9.6 Hz, 1 H, 4_C_), 3.91 (t, *J* = 10.3 Hz, 1 H, 6_C_), 3.77–3.74 (m, 2 H, 2_C_, 4_B_), 3.48–3.46 (m, 1 H, 3_C_), 3.36–3.32 (m, 1 H, 5_C_), 2.35 (s, 3 H, C*H*_3_), 1.43 (d, *J* = 6.18 Hz, 3 H, C-C*H*_3_). ^13^C NMR (125 MHz, CDCl_3_) δ 165.6 (*C*O), 138.5–125.5 (Ar-C), 103.6 (1_C_), 101.5 (PhCH), 85.9 (1_B_), 81.7 (4_B_), 78.4 (4_C_), 78.1 (3_C_), 78.0 (3_B_), 75.7 (2_C_), 75.5 (ArCH_2_), 75.2 (2_B_), 74.3 (OBn), 72.4 (OBn), 69.1 (5_B_), 68.5(6_C_), 67.7 (5_C_), 21.2 (C*H*_3_), 18.1 (C-C*H*_3_). ESI-HRMS [(M + Na)^+^] calcd for C_58_H_56_NaO_10_S: 967.3492; found: 967.3490.

### 3.6. Tolyl-[2-O-Benzyl-4,6-O-Benzylidene-β-D-Mannopyranosyl]-(1→3)-2-O-Benzoyl-4-O-Benzyl-1-Thio-α-L-Rhamnopyranoside (***6***)

To a solution of compound **14** (970 mg, 1.04 mmol) in CH_2_Cl_2_ (5 mL) was added an emulsion of DDQ [22] (354 mg, 1.56 mmol) in H_2_O (20 mL). The reaction mixture was allowed to stir at room temperature for 2 h. After 2 h, the reaction mixture was diluted with CH_2_Cl_2_ (100 mL), and the organic layer was washed with saturated aqueous solution of NaHCO_3_ (2 × 40 mL) and water (2 × 40 mL), dried over anhydrous Na_2_SO_4_, and concentrated. The crude product was purified by flash column chromatography on silica gel to give **6** (700 mg, 85%) as a white solid. *R_f_* = 0.4 (3:1 *n*-hexane/EtOAc); ^1^H NMR (600 MHz, CDCl_3_) δ 8.15–7.15 (m, 24 H, Ar-H), 5.69–5.67 (m, 1 H, 2_B_), 5.57 (d, *J* = 1.26 Hz, 1 H, 1_B_), 5.50 (s, 1 H, PhC*H*), 4.89–4.84 (m, 3 H, 1_C_, PhC*H*_2_), 4.78 (d, *J* = 11.16 Hz, 1 H, PhC*H*_2_), 4.48 (d, *J* = 11.46 Hz, 1 H, PhC*H*_2_), 4.37–4.34 (m, 2 H, 6_C_, 4_C_), 4.29–4.27 (m, 1 H, 3_B_), 3.83 (t, *J* = 10.3 Hz, 1 H, 6_C_), 3.79–3.75 (m, 3 H, 2_C_, 3_C_, 4_B_), 3.66–3.64 (m, 1 H, 5_B_), 3.37–3.33 (m, 1 H, 5_C_), 2.36 (s, 3 H, C*H*_3_), 1.44 (d, *J* = 6.18 Hz, 3 H, C-C*H*_3_). ^13^C NMR (125 MHz, CDCl_3_) δ 165.5 (*C*O), 138.0–126.3 (Ar-C), 103.5 (1_C_), 101.9 (PhCH), 85.9 (1_B_), 81.6 (4_B_), 79.1 (2_C_), 78.3 (3_C_), 78.0 (3_B_), 75.7 (2 OBn), 75.2 (6_B_), 75.1 (2_B_), 70.7 (5_B_), 69.2(4_C_), 68.5 (6_C_), 67.2 (5_C_) 21.2 (C*H*_3_), 18.1 (C-C*H*_3_). ESI-HRMS [(M + Na)^+^] calcd for C_47_H_48_NaO_10_S: 827.2866; found: 827.2860.

### 3.7. Tolyl-(2,3,4,6 Tetra-O-Acetyl)-α-D-Mannopyranosyl)-(1→2)-2-O-Benzyl-4,6-O-Benzylidene 1-Thio-α-D-Mannoyranoside (***5***)

To a solution of compound **8** [39] (217 mg, 0.45 mmol) and compound **7** [13] (0.29 g, 0.58 mmol) in anhydrous dichloromethane (20 mL), preactivated molecular sieves (MS-4 Å) were added. The reaction mixture was stirred at room temperature for 30 min under a nitrogen atmosphere. Then, the reaction mixture was cooled to −25 °C, and TMSOTf (30 μL) was added. It was allowed to warm to −15 °C and then stirred for 1 h. The reaction mixture was diluted with dichloromethane (50 mL), and the organic layer was washed with a saturated aqueous solution of NaHCO_3_ (40 mL) and water (40 mL) in succession, dried over anhydrous Na_2_SO_4_, filtered, and evaporated to dryness. The crude product was purified over a silica gel column to give pure disaccharide **5** (0.23 g, 66%) as a white fluffy solid. *R_f_* = 0.53 (3:2 *n*-hexane/EtOAc); ^1^H NMR (600 MHz, CDCl_3_) δ 7.63–7.15 (m, 14 H, Ar-H), 5.73 (s, 1 H, PhC*H*), 5.48–5.47 (m, 1 H, 2_E_), 5.46 (d, *J* = 1.14 Hz, 1 H, 1_D_), 5.42–5.40 (m, 1 H, 3_E_), 5.25 (t, *J* = 10.1 Hz, 1 H, 4_E_), 5.15 (brS, 1 H, 1_E_), 4.93 (d, *J* = 12.0 Hz, 1 H, PhC*H*_2_), 4.67 (d, *J* = 12.0 Hz, 1 H, PhC*H*_2_), 4.38–4.34 (m, 1 H, 4_D_), 4.27–4.23 (m, 3 H, 2_D_, 5_D_, 6_E_), 4.22 (m, 1 H, 6_E_), 4.07–4.01 (m, 3 H, 3_D_, 5_E_, 6_E_), 3.94 (t, *J* = 10.3 Hz, 1 H, 6_D_), 2.36 (s, 3 H, C*H*_3_), 2.14 (s, 3 H, COC*H*_3_), 2.01 (s, 3 H, COC*H*_3_), 2.02 (s, 3 H, COC*H*_3_), 1.96 (s, 3 H, COC*H*_3_). ^13^C NMR (125 MHz, CDCl_3_) δ 170.7 (COC*H*_3_), 169.9 (COC*H*_3_), 169.8 (COC*H*_3_), 169.7 (COC*H*_3_), 138.4–126.2 (Ar-C), 101.6 (PhCH), 99.7 (1E), 88.3 (1D), 79.2 (2D), 78.4 (5D), 75.8 (3D), 73.4 (OBn), 69.2 (2 C, 2E, 5E), 68.9 (3E), 68.4 (6D), 66.3 (4E), 65.2 (4D), 62.5 (6E), 21.1 (CO*C*H_3_), 20.9 (CO*C*H_3_), 20.7 (CO*C*H_3_), 20.5 (CO*C*H_3_). ESI-HRMS [(M + Na)^+^] calcd for C_41_H_46_NaO_14_S: 817.2506; found: 817.2510.

### 3.8. Tolyl-(2,3,4,6 Tetra-O-Acetyl)-α-D-Mannopyranosyl)-(1→2)-2-O-Benzyl-4,6-O-Benzylidene (1→3)-[2-O-Benzyl-4,6-O-Benzylidene-β-D-Mannopyranosyl]-(1→3)-2-O-Benzoyl-4-o-Benzyl-1-Thio-α-L-Rhamnopyranoside (***3***)

A mixture of disaccharide donor **5** (100 mg, 0.13 mmol), 1-(phenylsulfinyl)piperidine (BSP), (37.6 mg, 0.18 mmol, 1.4 equiv), 2,4,6-tri-*tert*-butylpyrimidine (TTBP), (65 mg, 0.26 mmol, 2 equiv), and 4 Å molecular sieves in CH_2_Cl_2_ (3 mL) was stirred under an atmosphere of nitrogen for 1 h [24]. The reaction was cooled to −60 °C and Tf_2_O (34 µL, 0.16 mmol, 1.2 equiv) was added. After 30 min of stirring at −60 °C, a solution of the acceptor **6** (152 mg, 0.20 mmol, 1.5 equiv) in CH_2_Cl_2_ (1 mL) was added using a cannula needle. The reaction mixture was stirred for a further 1 h at −60 °C and then quenched by the addition of triethyl phosphite [13] (32 µL, 0.20 mmol). The mixture was filtered, and the filtrate (100 mL) was washed with saturated NaHCO_3_ (30 mL) and brine (20 mL). The organic phase was dried over anhydrous Na_2_SO_4_, filtered, and the filtrate was concentrated under reduced pressure. The crude product was purified using flash column chromatography on silica gel (3:2 *n*-hexane/ethylacetate) to give **3** (102 mg, 55%) as a white fluffy solid. *R_f_* = 0.4 (3:2 *n*-hexane/ethylacetate); ^1^H NMR (600 MHz, CDCl_3_) δ 8.10–7.16 (m, 34 H, Ar-H), 5.71 (s, 1 H, PhC*H*), 5.69–5.68 (m, 1 H, 2_B_), 5.46 (d, *J* = 1.32 Hz, 1 H, 1_B_), 5.57–5.56 (m, 1 H, 2_E_), 5.52 (s, 1 H, PhC*H*), 5.35–5.33 (m, 1 H, 3_E_), 5.26 (t, *J* = 10.1 Hz, 1 H, 4_E_), 5.22 (d, *J* = 1.20 Hz, 1 H, 1_E_), 4.93 (d, *J* = 12.0 Hz, 1 H, PhC*H*_2_), 4.77–4.76 (m, 2 H, 1_C_, PhC*H*_2_), 4.68–4.60 (m, 4 H, 5E, 3 PhC*H*_2_), 4.41–4.26 (m, 5 H, 3_B_, 5_C_, 6_C_, 6_D_, 6_E_), 4.17 (t, *J* = 9.48 Hz, 1 H, 4_D_), 3.99–3.79 (m, 7 H, 1_D_, 3_C_, 4_B_, 5_B_, 6_C_, 6_D_, 6_E_), 3.76 (d, *J* = 2.34 Hz, 1 H, 2_D_), 3.69 (d, *J* = 2.94 Hz, 1 H, 2_C_), 3.37–3.31 (m, 2 H, 3_D_, 4_C_), 3.11–3.07 (m, 1 H, 5_D_), 2.36 (s, 3 H, C*H*_3_), 2.13 (s, 3 H, COC*H*_3_), 2.04 (s, 3 H, COC*H*_3_), 1.96 (s, 3 H, COC*H*_3_), 1.46 (d, *J* = 6.18 Hz, 3 H, C-C*H*_3_), 1.33 (s, 3 H, COC*H*_3_). ^13^C NMR (125 MHz, CDCl_3_) δ 171.0 (*C*OC*H*_3_), 170.1 (*C*OC*H*_3_), 170.1 (*C*OC*H*_3_), 170.0 (*C*OC*H*_3_), 165.7 (*C*O), 138.6–126.3 (Ar-C), 104.2 (1_C_), 101.7 (PhCH), 101.6 (PhCH), 99.6 (1_E_), 95.7 (1_D_), 86.2 (1_B_), 82.2, 79.5, 78.4, 77.9, 76.1, 75.9, 75.5, 75.4, 74.3, 73.9, 73.5, 72.3, 70.1, 69.7, 69.4, 68.8, 68.8, 68.6, 68.2, 67.6, 65.2, 62.0, 21.4 (C*H*_3_), 21.3 (CO*C*H_3_), 21.1 (CO*C*H_3_), 21.0 (CO*C*H_3_), 20.1(CO*C*H_3_), 18.4 (C-C*H*_3_). ESI-HRMS [(M + Na)^+^] calcd for C_81_H_86_NaO_24_S: 1497.5127; found: 1497.5150.

### 3.9. N-Succinimidyl-(2,3,4,6 Tetra-O-Acetyl)-α-D-Mannopyranosyl)-(1→2)-2-O-Benzyl-4,6-O-Benzylidene (1→3)-[2-O-Benzyl-4,6-O-Benzylidene-β-D-Mannopyranosyl]-(1→3)-2-O-Benzoyl-4-O-Benzyl-1-Thio-α-L-Rhamnopyranosyl]-(1→3)-2-O-Benzoyl-4,6-O-Benzylidene-β-D-Glucopyranoside (***2***)

To a solution of compound **3** (280 mg, 0.19 mmol) and compound **4** (44 mg, 0.38 mmol) in anhydrous CH_2_Cl_2_ (5 mL), preactivated 4 Å molecular sieves were added, and the reaction mixture was stirred under argon at room temperature for 45 min. The reaction mixture was cooled to −25 °C, and N-Iodosuccinimide (56 mg, 0.25 mmol) was added. After 10 min at the same temperature, trifluoromethane sulfonate (TMSOTf; 10 μL) was added, and the reaction mixture was stirred until completion of the reaction (1 h), as monitored by TLC (3:1 toluene/acetone). The reaction mixture was quenched with a saturated aqueous solution of NaHCO_3_ and aqueous 5% Na_2_S_2_O_3_ (2 × 20 mL), was filtered through a bed of Celite^®^-545, and the bed was washed with dichloromethane. The organic layer was washed 3x with aqueous NaHCO_3_ (40 mL) and water (3 × 20 mL), dried over anhydrous Na_2_SO_4_, filtered, and the filtrate was concentrated under reduced pressure to give a crude product. This crude material was purified using flash column chromatography on silica gel Acetone-Hexane (1:4) as the eluent to furnish pure β-compound **2** (87 mg, 61%) as a white fluffy solid. *R_f_* = 0.52 (3:1toluene/acetone); ^1^H NMR (600 MHz, CDCl_3_) δ 8.14–7.15 (m, 40 H, Ar-H), 5.74 (s, 1 H, PhC*H*), 5.69 (s, 1 H, PhC*H*), 5.62 (t, *J* = 4.98 Hz, 1 H, 2_A_), 5.55–5.54 (m, 1 H, 2_E_), 5.49 (s, 1 H, PhC*H*), 5.49–5.48 (m, 1 H, 1_A_), 5.43–5.42 (m, 1 H, 2_B_), 5.34–5.32 (m, 2 H, 1_B_, 3_E_), 5.23 (t, *J* = 10.3 Hz, 1 H, 4_E_), 5.20 (d, *J* = 1.08 Hz, 1 H, 1_E_), 4.90 (d, *J* = 12.1 Hz, 1 H, PhC*H*_2_), 4.77 (d, *J* = 11.8 Hz, 1 H, PhC*H*_2_), 4.72 (s, 1 H, 1_C_), 4.70–4.55 (m, 6 H, 4_A_, 5_E_, 4 PhC*H*_2_), 4.39–4.12 (m, 8 H, 3_A_, 6_A_, 3_B_, 5_C_, 6_C_, 4_D_, 6_D_, 6_E_), 4.00–3.62 (m, 11 H, 5_A_, 6_A_, 4_B_, 5_B_, 2_C_, 3_C_, 6_C_, 1_D_, 2_D_, 6_D_, 6_E_), 3.35–3.32 (m, 1 H, 3_D_), 3.30–3.27 (m, 1 H, 4_C_), 3.07–3.03 (m, 1 H, 5_D_), 2.76 (s, 4 H, succinimide), 2.12 (s, 3 H, COC*H*_3_), 2.02 (s, 3 H, COC*H*_3_), 1.95 (s, 3 H, COC*H*_3_), 1.31 (s, 3 H, COC*H*_3_), 1.07 (d, *J* = 6.18 Hz, 3 H, C-C*H*_3_). ^13^C NMR (125 MHz, CDCl_3_) δ 170.7 (*C*OC*H*_3_), 170.6 (*C*OC*H*_3_), 169.8 (2 C, -C(O)CH_2_-CH_2_-C(O)), 169.7 (*C*OC*H*_3_), 164.9 (*C*O), 164.8 (*C*O), 138.4–125.3 (Ar-C), 103.8 (1_C_), 102.6 (1_A_), 101.5 (PhCH), 101.3 (PhCH), 100.3 (PhCH), 99.3 (1_E_), 96.7 (1_B_), 95.3 (1_D_), 81.4, 79.2, 78.0, 77.7, 77.6, 76.0, 75.9, 75.5, 74.8, 74.0, 73.5, 73.2, 72.9, 72.2, 72.1, 69.8, 69.4, 68.5, 68.5, 68.4, 68.3, 67.8, 67.8, 67.3, 66.3, 65.0, 61.8, 25.5 (succinimide), 21.0 (CO*C*H_3_), 20.8 (CO*C*H_3_), 20.7 (CO*C*H_3_), 19.8 (CO*C*H_3_), 17.6 (C-C*H*_3_). ESI-LRMS [(M + Na)^+^] calcd for C_98_H_101_NaNO_33_: 1842.6; found: 1842.1.

### 3.10. Aminooxy-(α-D-Mannopyranosyl)-(1→2)-(β-D-Mannopyranosyl)-(1→3)-(β-D-Mannopyranosyl)-(1→3)-(α-L-Rhamnopyranosyl)-(1→3)-β-D-Glucopyranoside (***1***)

Compound **2** (30 mg, 0.016 mmol) was dissolved in a mixture of methanol/ethylacetate (4:1) 20 mL followed by the addition of 10% Pd/C (70 mg, 0.659 mmol). The mixture was stirred at room temperature under an H_2_ atmosphere (1 atm). After 24 h, a drop of 12 M hydrochloric acid was added and allowed to stir for an additional 6 h. The reaction was monitored by ESI mass spectrophotometry. After completion, the solution was then filtered through a filter paper to remove Pd/C, and the filtrate was concentrated down by air purging after which the crude product was dissolved in a mixture of MeOH: H_2_O (5:1), respectively. Hydrazine monohydrate (33 mg, 0.66 mmol, 40 eq) was added, and the resulting mixture was allowed to stir overnight for 24 h. When the reaction was completed, the mixture was concentrated and purified by bio gel P-2 column chromatography (H_2_O) and lyophilized to afford compound **1** (8 mg, 59% over two steps) as a colorless solid. ^1^H NMR (600 MHz, D_2_O) δ 5.17 (s, 2H), 4.85 (d, *J* = 3.8 Hz, 3H 1_c_,1_D_), 4.28 (d, 2H), 4.20 (d, 2H), 4.10–4.02 (m, 2H), 3.96–3.83 (m, 8H), 3.79–3.74 (m, 3H), 3.74–3.53 (m, 10H), 3.53–3.49 (m, 1H), 3.41–3.34 (m, 3H), 1.24 (d, *J* = 6.2 Hz, 3H C-C*H*_3_). ^13^C NMR (151 MHz, D_2_O) δ 105.12, 101.16, 101.10, 100.62, 96.58, 81.72, 79.32, 79.02, 76.82, 76.21, 76.04, 75.60, 73.58, 72.24, 71.99, 71.02, 70.24, 70.14, 69.95, 68.81, 67.62, 67.57, 66.84, 66.49, 64.96, 60.97, 60.67, 60.46, 16.42. ESI-HRMS [(M + H)^+^] calcd for C_30_H_53_NaNO_25_: 828.2985; found: 828.3070.

## 4. Synthesis of Psl-Conjugates

### 4.1. Methoxy-Squaramido-(α-D-Mannopyranosyl)-(1→2)-(β-D-Mannopyranosyl)-(1→3)-(β-D-Mannopyranosyl)-(1→3)-(α-L-Rhamnopyranosyl)-(1→3)-β-D-Glucopyranoside (***15***)

Dimethyl squarate (1 mg, 0.001 mmol) was first dissolved in a small amount of DMSO (10 µL). This solution was then diluted with 30 µL of PBS buffer (phosphate-buffered saline) [29]. In a separate vial, compound **1** (3.4 mg, 0.02 mmol) was dissolved in 60 µL of PBS buffer. The squarate solution was then transferred to the vial containing compound **1**, and the pH was adjusted to 7.2 using 2 M NaOH. The resulting solution was incubated at 37 °C for 2 h at 220 rpm agitation to allow the reaction to proceed. After the reaction, the product was purified by size exclusion chromatography using a P2 gel column (5 mL) to give compound **15** (1.03 mg, 94%). The ESI-MS data provided (see Figure S13) confirm the molecular weight of the purified compound **15**. ESI-LRMS [(M + Na)^+^] calcd for C_35_H_57_NaNO_28_: 960.3; found: 960.4.

### 4.2. Synthesis of Psl-CRM197 Conjugate (**16**)

In a 1.5 mL Eppendorf tube, 250 µL of 200 mM PBS (pH 9.5) was used to dissolve commercial-grade CRM197 (1 mg, 17 µmol), and 150 µL of 0.2% glucose solution was added [30]. Methoxy-squarate functionalized glycan (0.069 mmol, 4 eq) was dissolved. The two solutions were mixed and allowed to incubate at room temperature overnight. To count the number of glycans added to CRM197, the reaction was tracked using MALDI-TOF-MS. Product **16** was purified using an Amicon Ultra 3k MWCO centrifugal filter (MilliporeSigma, Burlington, MA, USA) to concentrate and desalt the sample. The desired conjugate **16** was confirmed by SDS-PAGE and MALDI-TOF (see Figure S15).

### 4.3. Synthesis of Psl-Biotin Conjugate for ELISA Coating (***18***)

Commercially available BiotinPEG3aldehyde (1.0 mg, 0.0018 mmol) was dissolved in 20 μL of dimethyl sulfoxide. A total of 2.5 equivalents of compound **1** (4 mg, 0.0045 mmol) were added to 1× sodium acetate buffer. Additionally, a 10 mM concentration of 4-aminophenol was introduced at pH 5.0. The resulting reaction mixture was stirred in the dark under an inert nitrogen atmosphere. ESI mass spectrometry was employed to monitor the progress of the reaction. After 48 h, the reaction appeared to be complete. It was then quenched by adding hydroxylamine. The crude product was purified by size exclusion chromatography column (P2 gel, 5 cm length), and fractions were checked with the charring method (5% H_2_SO_4_ in methanol). ESI mass spectrometry was used in the characterization of purified product **18** (1.5 mg, 63%). ESI-LRMS [(M + Na)^+^] calcd for C_56_H_89_NaN_5_O_3_1S: 1382.5; found: 1382.3.

### 4.4. Mice and Vaccination

Six- to seven-week-old female C57BL/6 (B6) mice were obtained from Jackson Laboratory, (Bar Harbor, ME, USA). The mice were housed in the Department of Laboratory Animal Resources at the University of Toledo in specific pathogen-free housing, according to the National Institutes of Health guidelines, with oversight by the University of Toledo Institutional Animal Care and Use Committee, protocol 400049, latest approval 4/7/2023. Groups of 4 mice were immunized intraperitoneally with 100 µL vaccine or adjuvant alone. The mice were boosted on days 14 and 21 after the initial immunization. The serum was collected using a submandibular needle prior to and in between the vaccinations in BD Microtainer tubes (cat # 365967) from Thermo Fisher Scientific (Waltham, MA, USA). The collected blood samples were centrifuged at 15,000 rpm for 5 min at room temperature. After centrifugation, the serum was carefully transferred to new tubes, divided into smaller aliquots, and stored at 4 °C until further use.

### 4.5. Spleen Cell Isolation

The mice were euthanized according to the NIH guidelines, and the spleens were isolated according to a previously published protocol [40]. Spleen cells were suspended in T cell medium, which was composed of Roswell Park Memorial Institute (RPMI)-1640 with 116 μg/mL arginine, 36 μg/mL asparagine, 216 μg/mL glutamine, 110 μg/mL pyruvate, 6 μg/mL folic acid, 10% FBS (Atlanta Biological, cat # S11195H, Bio Techne, Minneapolis, MN, USA), 10 mM *N*-(2-hydroxyethyl)piperazine-N′-ethanesulfonic acid (HEPES), 1000 U/mL penicillin, 100 μg/mL streptomycin, and 0.05 mM β-mercaptoethanol. Red Cell Lysing Buffer (cat # R7757, Sigma-Aldrich, St. Louis, MO, USA) was added to remove erythrocytes in the cell suspension, followed by centrifugation at 1500 rpm for 5 min. The resulting cell pellet was resuspended in media, and this washing step was repeated for two more times. Finally, the cells were filtered through a 70 μm nylon mesh cell strainer (Fisher Scientific, cat # 22363548, Thermo Fisher Scientific, Waltham, MA, USA) and counted using trypan blue dye.

### 4.6. ELISA

A high-binding Immulon 4HBX 96-well plate (Fisher Scientific cat # 14-245-153, Thermo Fisher Scientific, Waltham, MA, USA) was coated with 0.2 µg/mL Psl-CRM197 antigen or 0.2 µg/mL Psl-biotin. Psl-biotin was coated on a plate pre-coated with 0.2 mg/mL streptavidin. The plate was washed three times with PBS buffer containing 0.05% Tween 20 (PBS-T). Next, the plate was blocked with PBS-B, which consists of 1% bovine serum albumin (BSA) fraction V for RIA and ELISA (Sigma-Aldrich, St. Louis, MO, USA, cat # 126593-25GM), in PBS for 1 h. Then plate was washed three times with PBS-T and incubated with mice serum at different dilutions. After incubating the serum for 1 h, the plate was washed again five times, followed by the incubation of the peroxidase goat anti-mouse IgG1 + IgG2a + IgG2b + IgG3 (Jackson ImmunoResearch, West Grove, PA, USA, cat # 115-035-164) secondary antibody at 1:5000, diluted in 0.1% BSA in PBS buffer. Finally, the plate was washed again and developed using BioFX 3,3′,5,5′-tetramethylbenzidine-horseradish peroxidase (TMB-HRP) microwell substrate (Surmodics IVD, Inc., Eden Prairie, MN, USA, cat # TMBW-1000-01). The absorbance readings were taken at a visible wavelength 600 nm in a ClarioStar plate reader (Danaher Corp., Washington, DC, USA). In the isotype-specific ELISA, the initial steps were identical, except for the substitution of the secondary antibody. Here, goat anti-mouse IgG1, IgG2a, and IgG3 polyclonal antibodies from Thermo Fisher were used for detection.

### 4.7. Whole Cell ELISA Assay

*P. aeruginosa* strain PAO1 was obtained from Dr. Erin Prestwich’s laboratory at the University of Toledo. To determine the binding of antibodies in the mice serum to intact bacteria, whole cell ELISA assay was performed according to a previously published method [32]. Briefly, an overnight culture of bacteria was washed and diluted with LB media (Fisher Scientific) to an OD_600_ = 0.6. An Immulon 4HBX 96 -well plate was coated with the diluted bacteria culture for 2 h at room temperature. After the incubation, the plate was washed three times with PBS-T, followed by blocking with PBS-B for 1 h. The plate was washed again and incubated for 1 h with different dilutions of mouse serum. The plate was washed again five times with PBS-T, and peroxidase goat anti-mouse IgG1 + IgG2a + IgG2b+ IgG3 (Jackson ImmunoResearch, cat # 115-035-164) secondary antibody was added for 1 h. The plate was washed five times to remove any unbound antibodies and developed with TMB substrate (Surmodics IVD). The absorbance readings were read at 600 nm using a ClarioStar microplate reader.

### 4.8. Opsonophagocytic Killing Assay

The opsonophagocytic killing assay was used to determine the complement- and macrophage-mediated killing of bacteria in the presence of antibodies in the serum. A log-phase bacteria culture at OD_600_ = 0.3 was diluted to 1:100 in LB media. An aliquot of 10 µL of the bacterial culture was incubated for 1 h with 20 µL of mouse serum at 1:10 dilution or with media for control. After incubation, 10 µL of the bacteria–antibody mixture was mixed with 50 µL of 1 × 10^6^ mouse macrophage RAW264.7 cells (ATCC, Manassas, VA, USA), with standard rabbit complement (Cedarlane, Burlington, ON, Canada) at a 1:5 final volume. The control sample received 50 µL of media. This was incubated again for 1 h with shaking at 37 °C. Following incubation, each sample was diluted appropriately in DMEM, 10% FBS, and plated on LB agar for bacterial counts [32]. The following equation was used to calculate the killing percentage:

$$\%\;killing=\frac{CFU\;surviving\;in\;control\;sample-CFU\;surviving\;in\;test\;sample}{CFU\;surviving\;in\;control\;sample}$$

### 4.9. Cell Proliferation Using the MTT Assay

Aliquots of spleen cells were added to a 96-well plate so that each well contained 4 × 10^5^ spleenocytes. The cells were incubated with CRM197-Psl antigen at concentrations 0.001, 0.01, 0.1, 1, 10, 100 nM, and 1 μM for 6 days at 37 °C and under 5% of CO_2_. After the incubation, the media was aspirated, and with 200 μL of the HBSS buffer (Gibco cat # 14025-092, Thermo Fisher Scientific, Waltham, MA, USA) was added to each well. The plate was then developed with 20 μL of MTT substrate (Promega G4000, Madison, WI, USA). The reaction was stopped by adding 30 μL of the Promega stopping buffer, and the absorbance reading was taken at 570 nm using a ClarioStar plate reader.

### 4.10. Single-Color Murine IgG ELISPOT

The spleen cells obtained from vaccinated mice were activated using Mouse B-Poly-S (ImmunoSpot, CTL, Shaker Heights, OH, USA) and cultured at 37 °C with 5–10% CO_2_ for 3–5 days to stimulate the B-cells. After stimulation, the cells were plated onto 96-well plates coated with anti-IgG antibody from the Mouse IgG Single-Color ELISPOT kit by ImmunoSpot^®^, with a cell density of 1 × 10^6^ cells/mL per well. The subsequent procedures were performed following the manufacturer’s instructions. Finally, the readings were measured using a CTL ImmunoSpot^®^ S6 Entry M2 Analyzer.

### 4.11. Double-Color Murine IFN-γ/IL-17 ELISPOT

IFN-γ/IL-17 cytokine ELISPOT was performed using the Double-Color Murine IFN-γ/IL-17 ELISPOT kit by ImmunoSpot^®^. Spleen cells at 1 × 10^6^ cells/mL per well were co-cultured with different concentrations of Psl-CRM197 or concanavalin A (ConA) onto a 96-well plate pre-coated with IFN-γ/IL-17 capture antibodies. The cells were incubated overnight. The detection and development of the plate were performed according to the manufacturer’s instructions. The plate was analyzed using the CTL ImmunoSpot^®^ S6 Entry M2 Analyzer.

## 5. Statistics

The vaccine data shown are representative or averages of 3 separate experiments. Data were plotted and analyzed using GraphPad Prism version 10.1.2. Where statistical comparisons were conducted, the groups were compared using one-way ANOVA with a Tukey multiple comparisons post-test. * = *p* < 0.05. A group size of *n* = 4 was determined based on a power analysis of prior similar data using StatMate 2.0 software, which revealed that *n* = 4 would provide 80% power to detect a difference in means of 0.28 with an alpha equal to 0.05.

## 6. Conclusions

In conclusion, we have successfully synthesized the aminooxy derivative of the pentasaccharide-repeating unit of *Pseudomonas aeruginosa* Psl exopolysaccharide. This pentasaccharide was prepared efficiently in 23 steps utilizing [2 + 2 +1] block chemical synthesis with good anomeric selectivity and subsequently reduced the number of reaction steps compared to the previous Psl syntheses. The aminooxy linkage at the reducing end of the Psl repeat was further linked to CRM197 to give a potential glycoconjugate vaccine candidate for PA. We demonstrated that the conjugate vaccine could generate anti-Psl antibodies that could recognize PAO1 bacteria and initiate the opsonophagocytic killing of the bacteria.

## Figures and Tables

**Table 1:** Composition of QS-21 liposomes for 1 mL solution.

Formulation	DPMG	DMPC	Cholesterol	Pam_3_CysSK4	QS-21
mol %	4.4	40	54.2	1.5	
Amount (mg)	0.909	8.135	6.288	0.733	0.010
Molecular weight (g/mol)	688.8	677.9	386.7	1631	1990

**Table 2:** Summary of the two sets of samples prepared for vaccination (1 mL solution).

Samples	DPMG (mg)	DMPC (mg)	Cholesterol (mg)	Pam_3_CysSK_4_ (mg)	QS-21 (mg)	16 (mg)
QS-21 liposome (Control)	0.909	8.135	6.288	0.733	0.01	-
QS-21 + Psl-CRM197 liposome	0.909	8.135	6.288	0.733	0.01	0.28

## Data Availability

The original contributions presented in this study are included in the article/supplementary material. Further inquiries can be directed to the corresponding authors.

## Supplementary Materials

The following supporting information can be downloaded at: https://www.mdpi.com/article/10.3390/molecules30081720/s1. Supplementary information. ^1^H and ^13^C NMR spectra of compounds **1–6**, **9–10**, **12d**, **14**, ESI-MS spectra of compounds **15** and **18**, PAGE of target proteins, MALDI-TOF of compounds **16** and **17**, DLS characterization of liposomes.
