# Supplementary material for: Synthesis of the Pentasaccharide Unit of the Pseudomonas aeruginosa Exopolysaccharide Psl Conjugation with CRM197, and Evaluation of Antigenicity in a QS-21/Pam3CSK4-Liposomal Formulation
Source: Molecules. 2025 Apr 11;30(8):1720. doi: 10.3390/molecules30081720 (PMC12029197; doi:10.3390/molecules30081720)

Supporting Information

Synthesis of the pentasaccharide unit of the *Pseudomonas aeruginosa* exopolysaccharide Psl conjugation with CRM197, and evaluation of antigenicity in a QS-21/Pam_3CSK_4-liposomal formulation

Uzoamaka Clara Bokolo[1†], Ravindika Dissanayake[2†], Samir Ghosh[1†], Shadia Nada[2], Babatunde S. Obadawo[1], Erin G. Prestwich[2], Katherine A. Wall[2] and Steven J. Sucheck[1*]*

[1]Department of Chemistry and Biochemistry, The University of Toledo, 2801 W. Bancroft Street, Toledo, OH 43606, United States

[2]Department of Medicinal and Biological Chemistry, The University of Toledo, 3000 Arlington Ave., Toledo, OH 43614, United States

Table of Contents Page No.

Paper Title, author's names, and school address	S1
^{1}H and ^{13}C NMR spectra of the synthesized compounds	S3-S13
HRMS and ESI Mass Spec of compounds	S14-S17
SDS PAGE of conjugate **16**	S18
MALDI-TOF data of compound **16**	S19
SDS-PAGE of conjugate **17**	S19
MALDI-TOF of conjugate **17**	S20
DLS data on liposomes	S21-S23

Spectral Data

Figure S1: ^{1}H and ^{13}C NMR of compound **12d** ($CDCl_3$, 600 MHz)

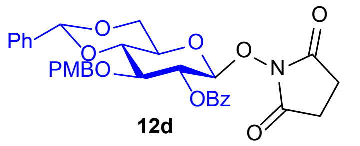

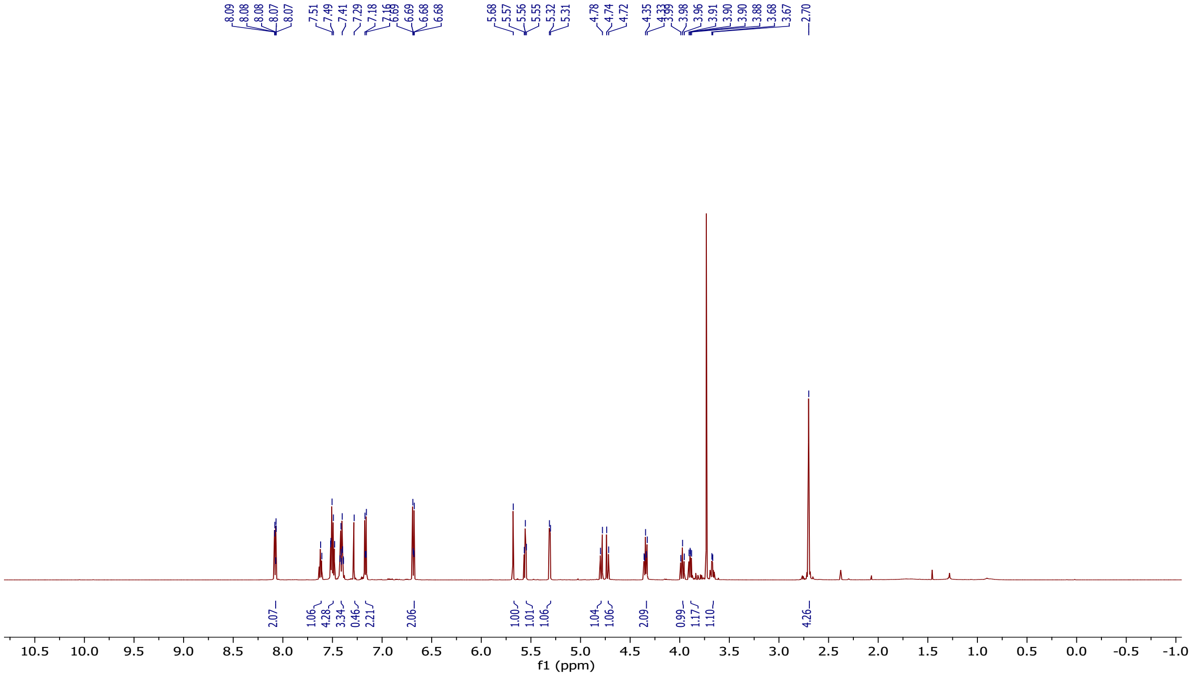

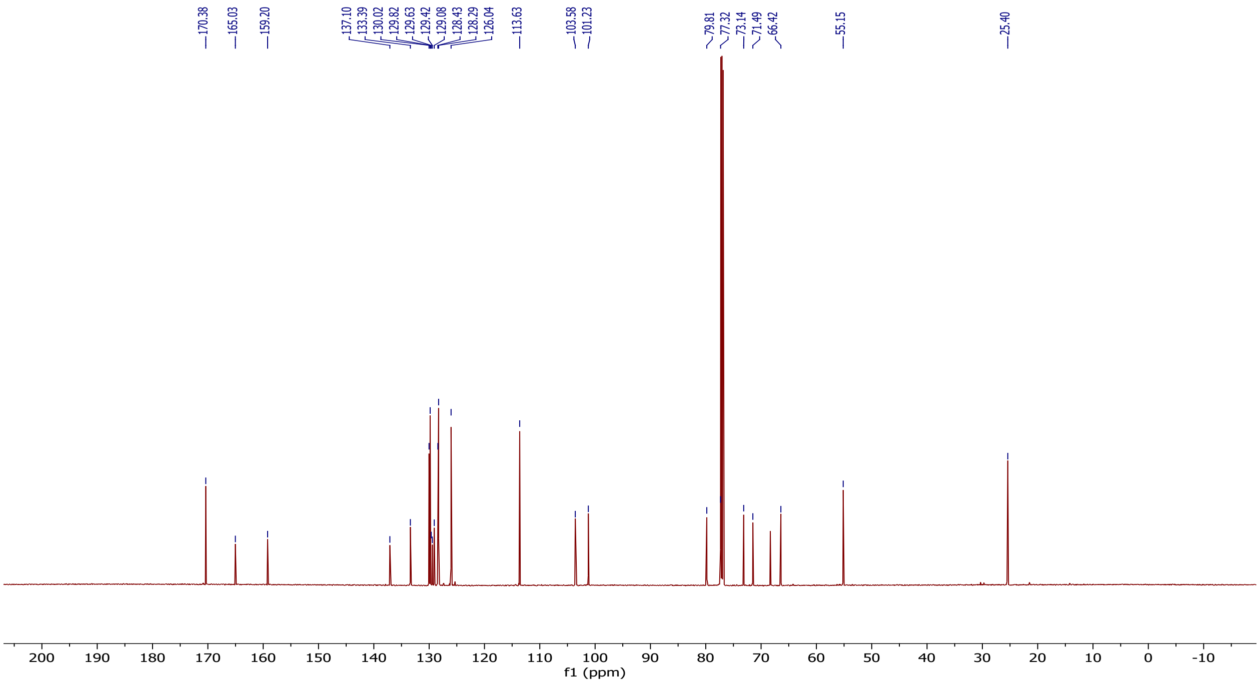

Figure S2: ^{1}H and ^{13}C NMR of compound **4** (CDCl$_3$, 600 MHz)

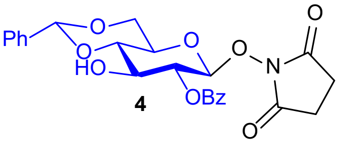

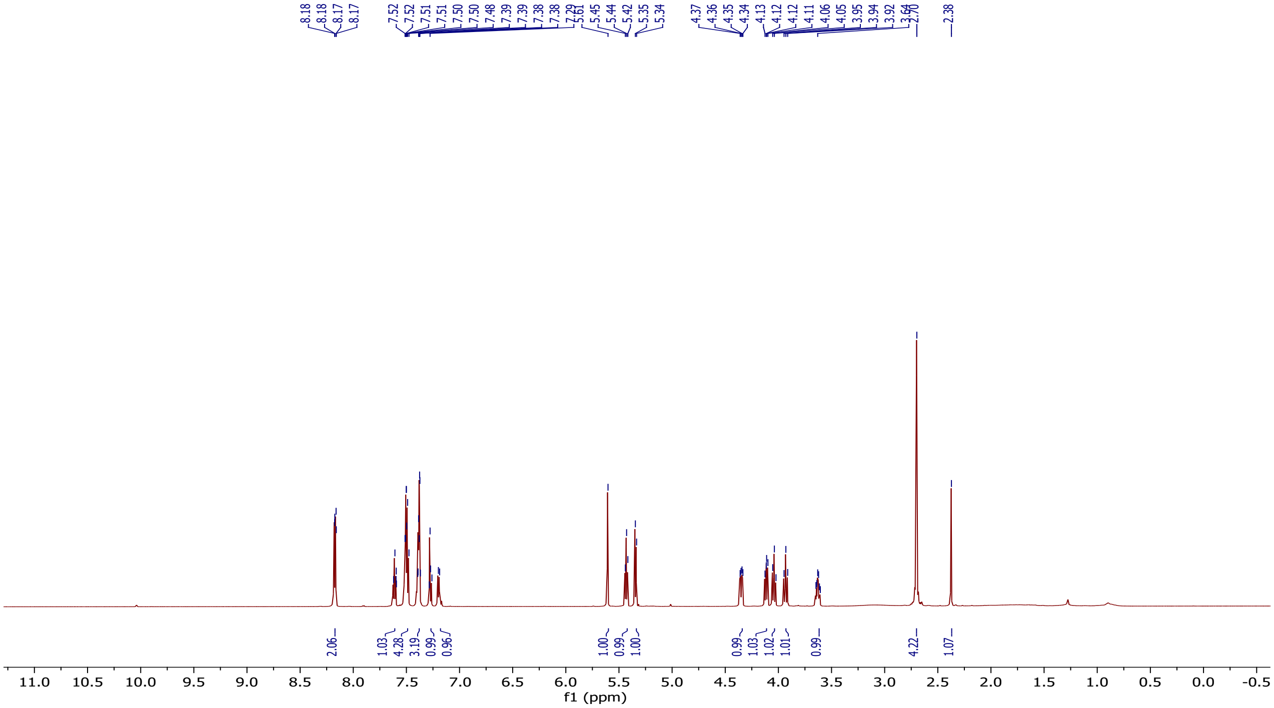

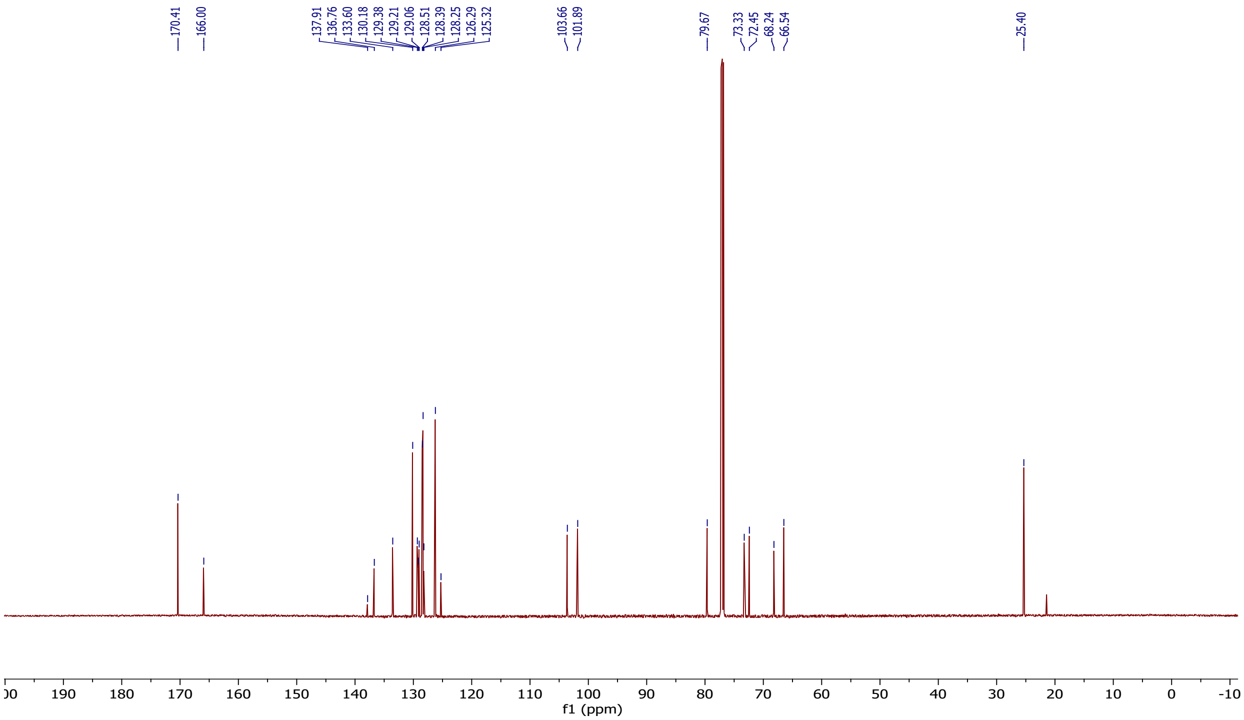

Figure S3: ^{1}H and ^{13}C NMR of compound **10** ($CDCl_3$, 600 MHz)

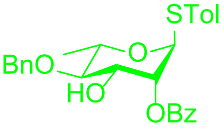

10

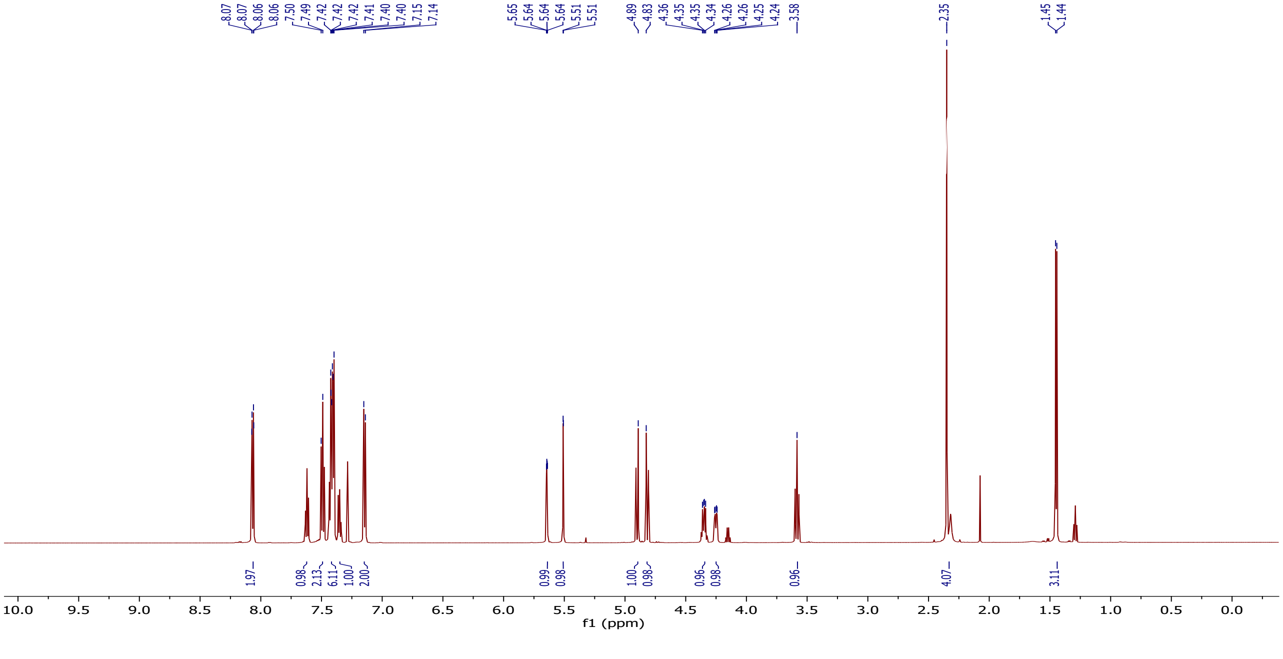

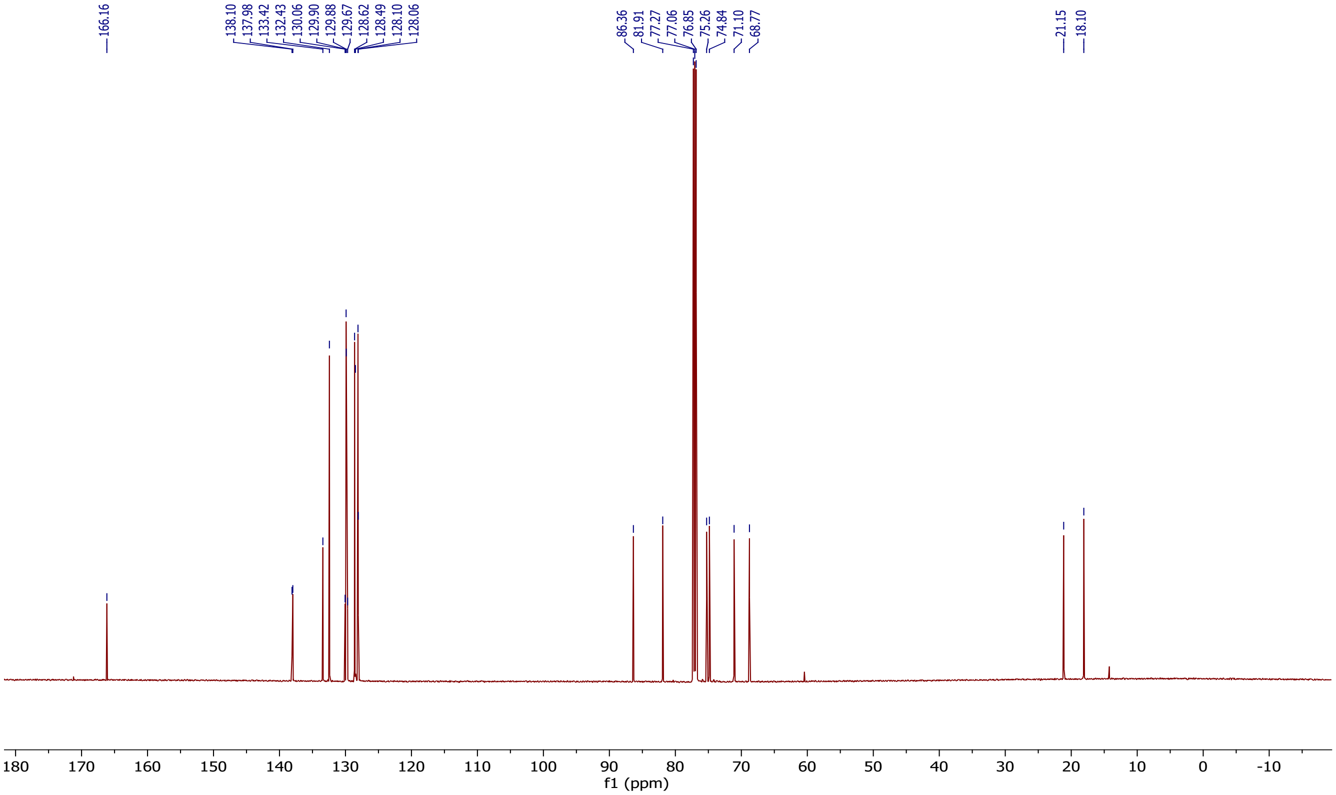

Figure S4: ^{1}H and ^{13}C NMR of compound **13 (**$CDCl_3$, 600 MHz)

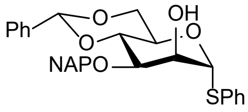

13

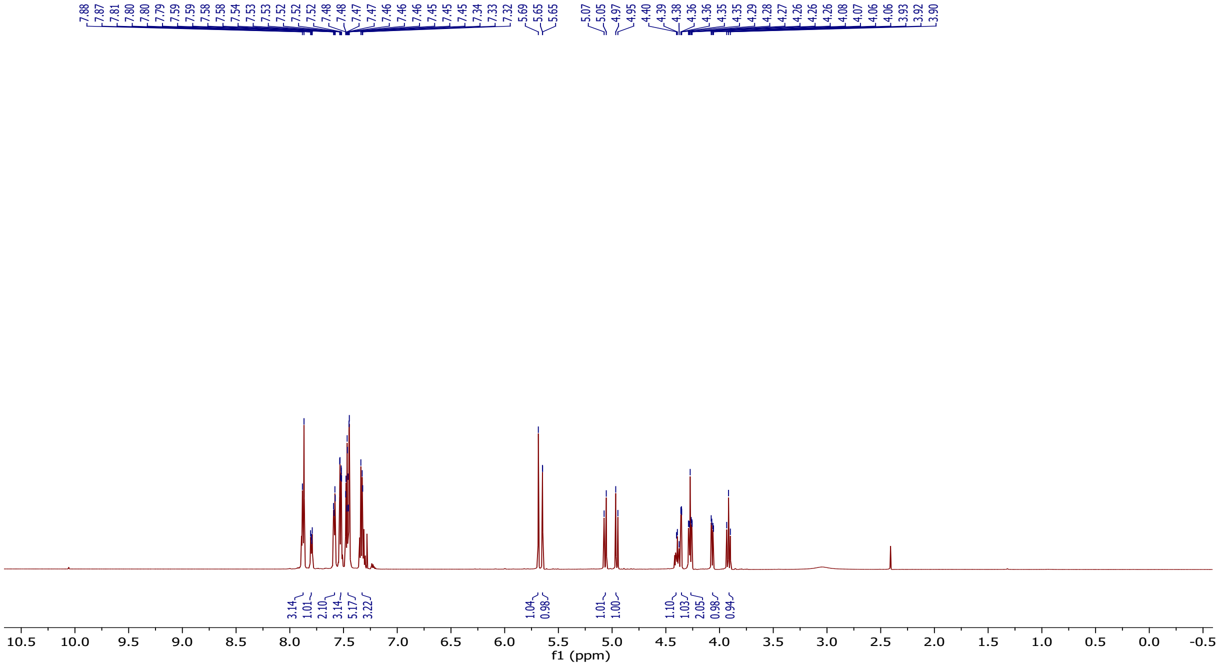

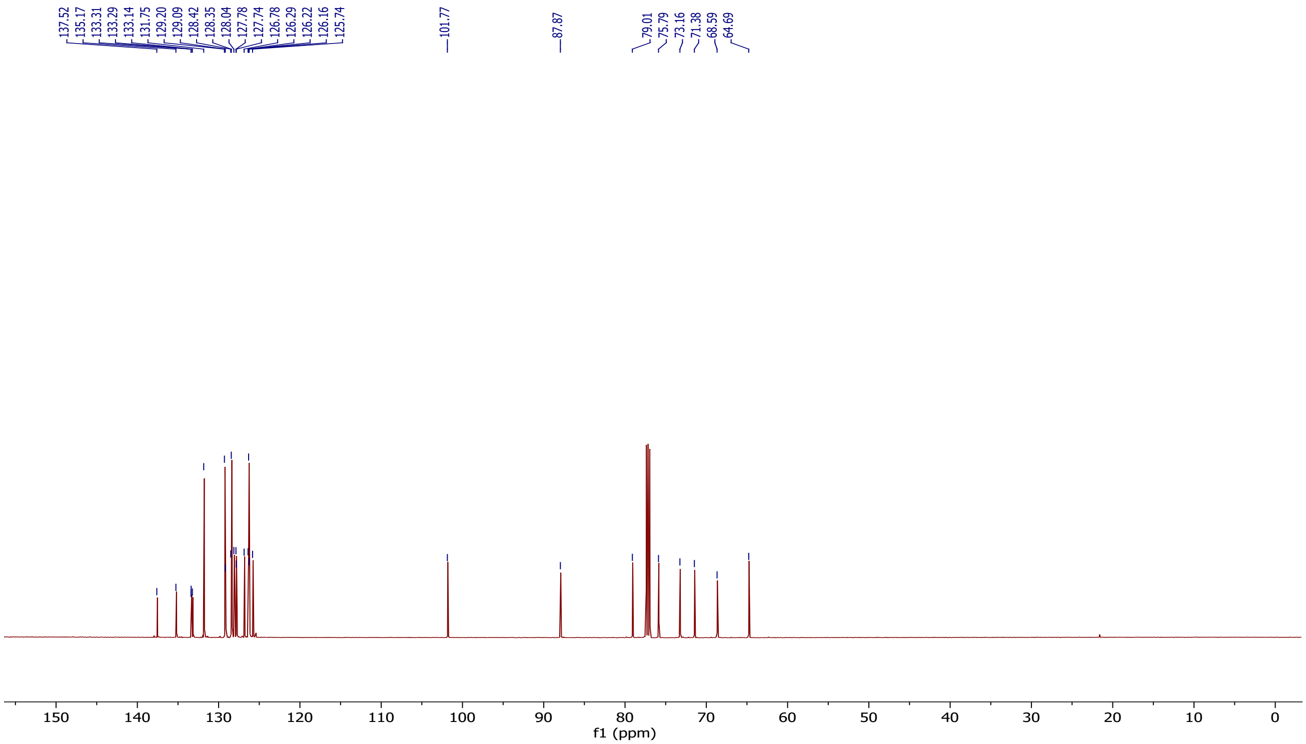

Figure S5: ^{1}H and ^{13}C NMR of compound **9** (CDCl$_3$, 600 MHz)

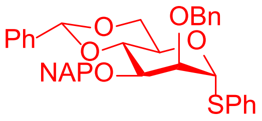

9

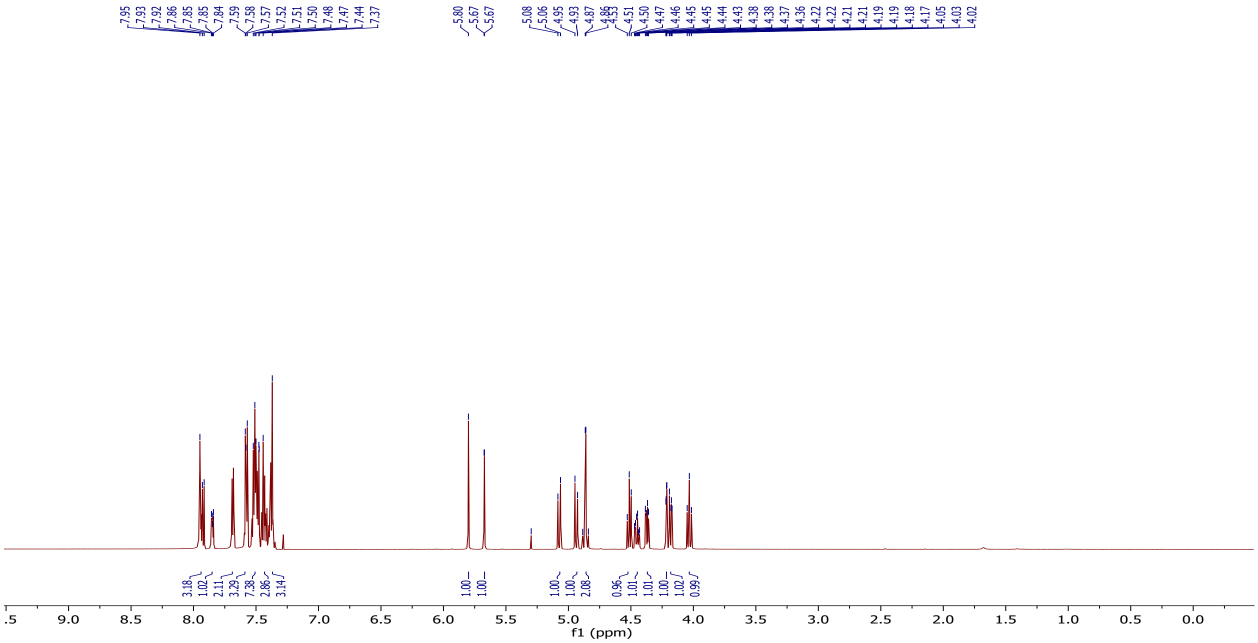

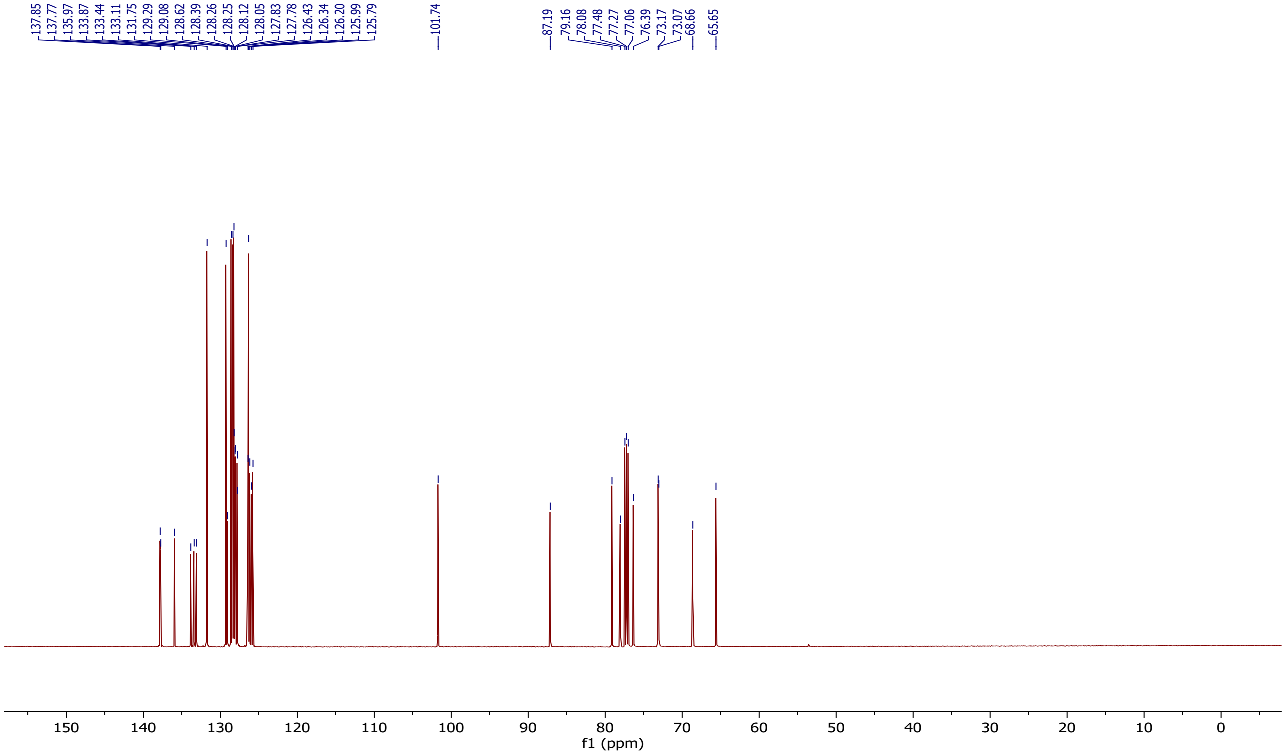

Figure S6: ^{1}H and ^{13}C NMR of compound **14** ($CDCl_3$, 600 MHz)

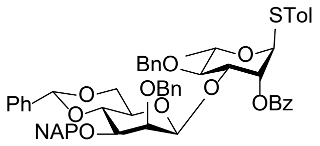

14

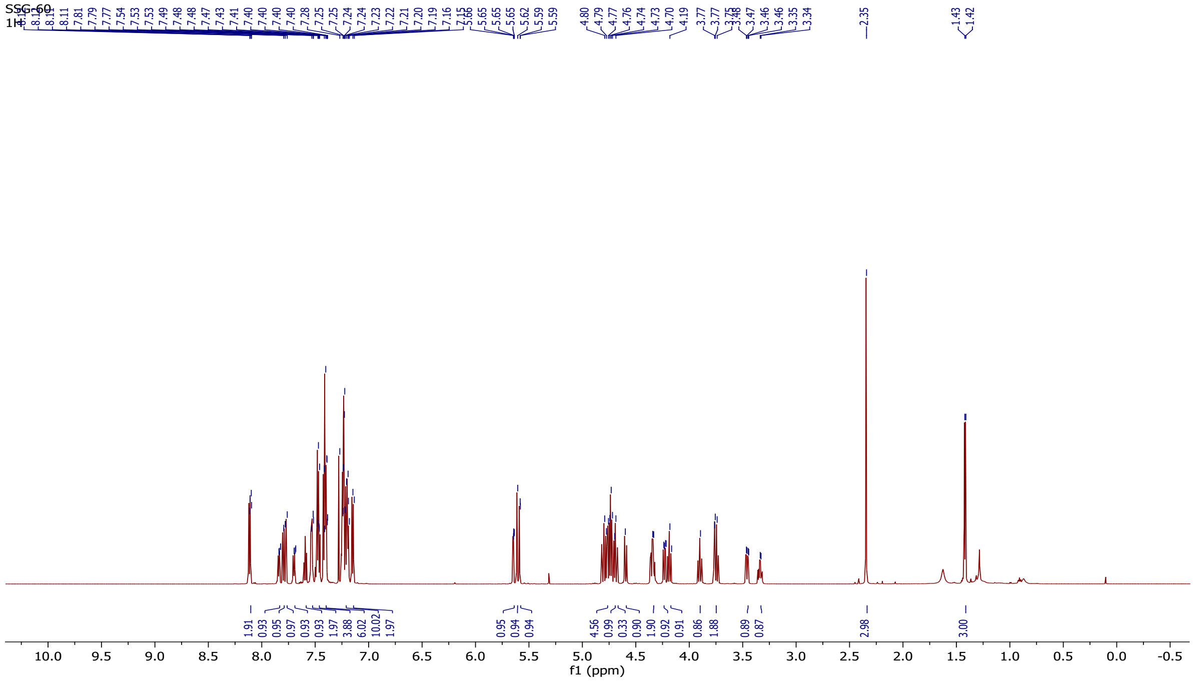

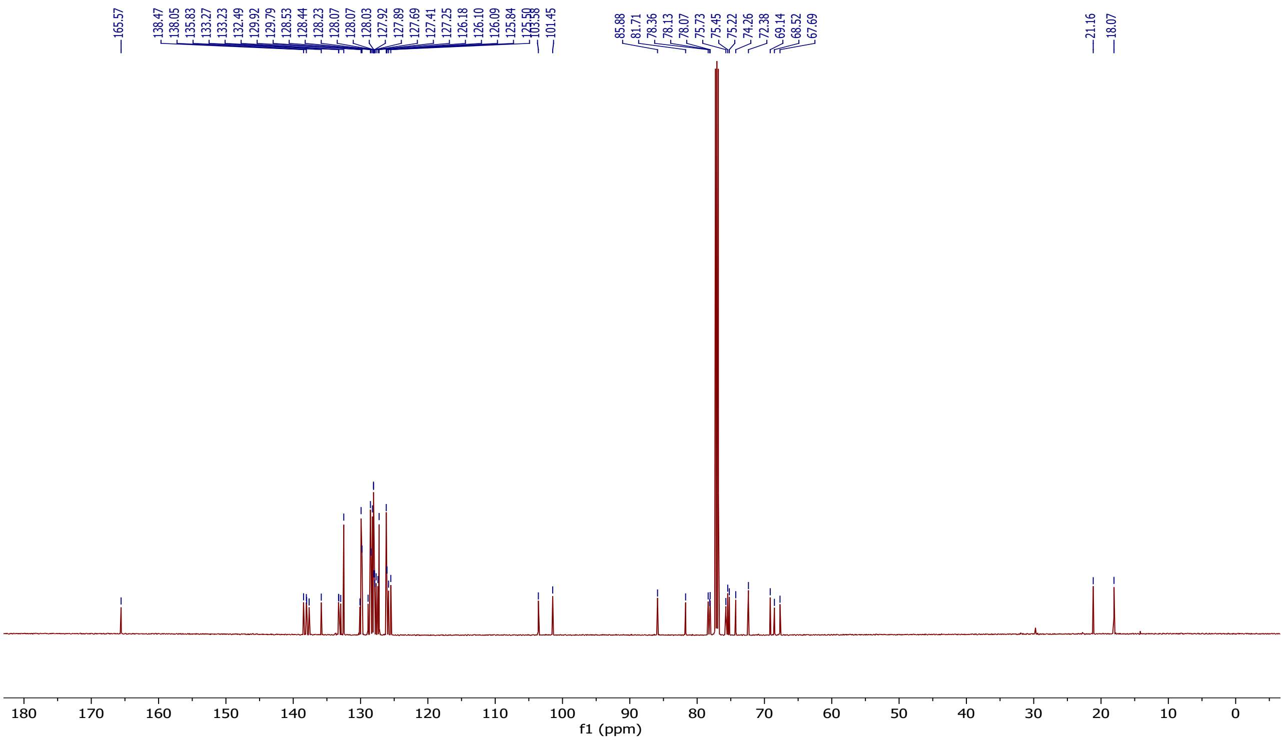

Figure S7: ^{11}H and ^{13}C NMR of Compound **6** ($CDCl_3$, 600 MHz)

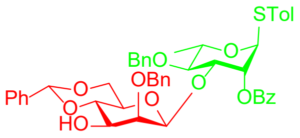

6

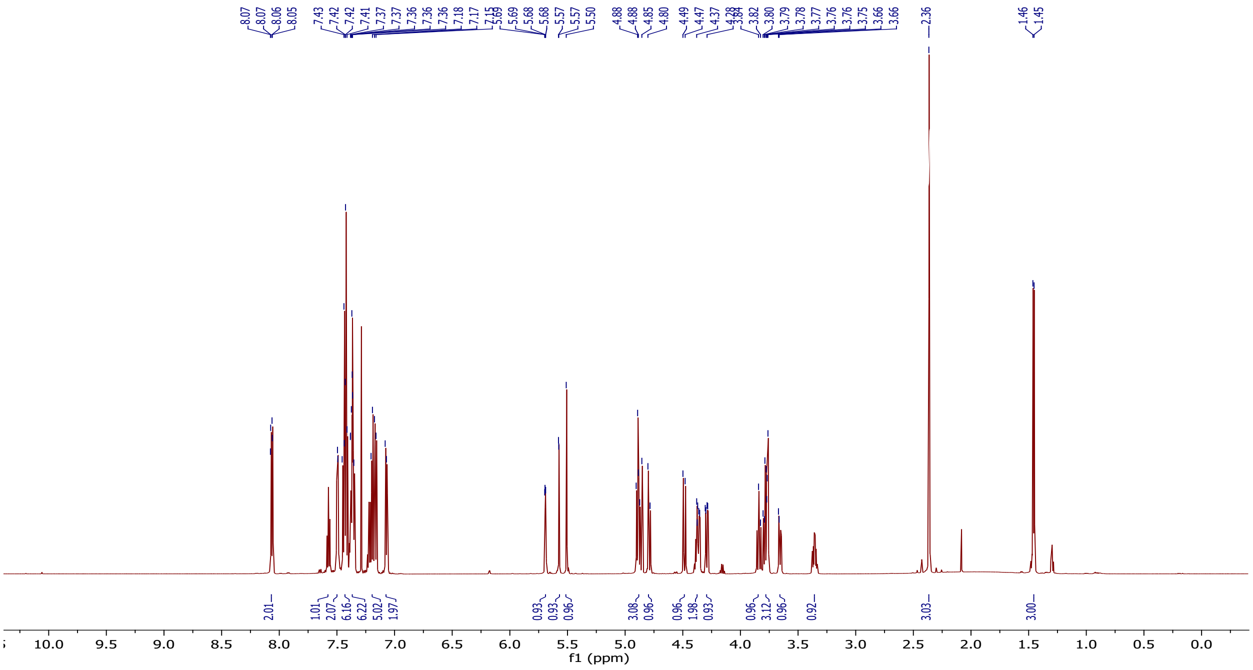

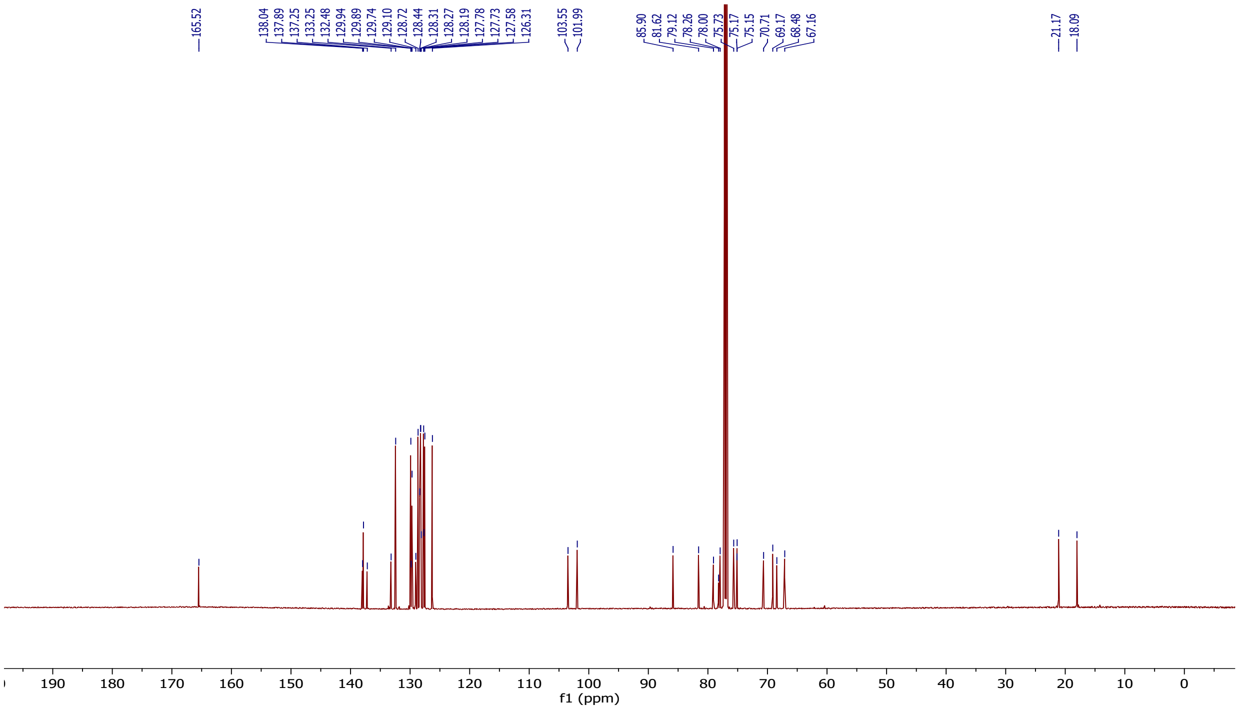

Figure S8: ^{1}H and ^{13}C NMR of Compound **5** ($CDCl_3$, 600 MHz)

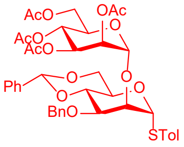

5

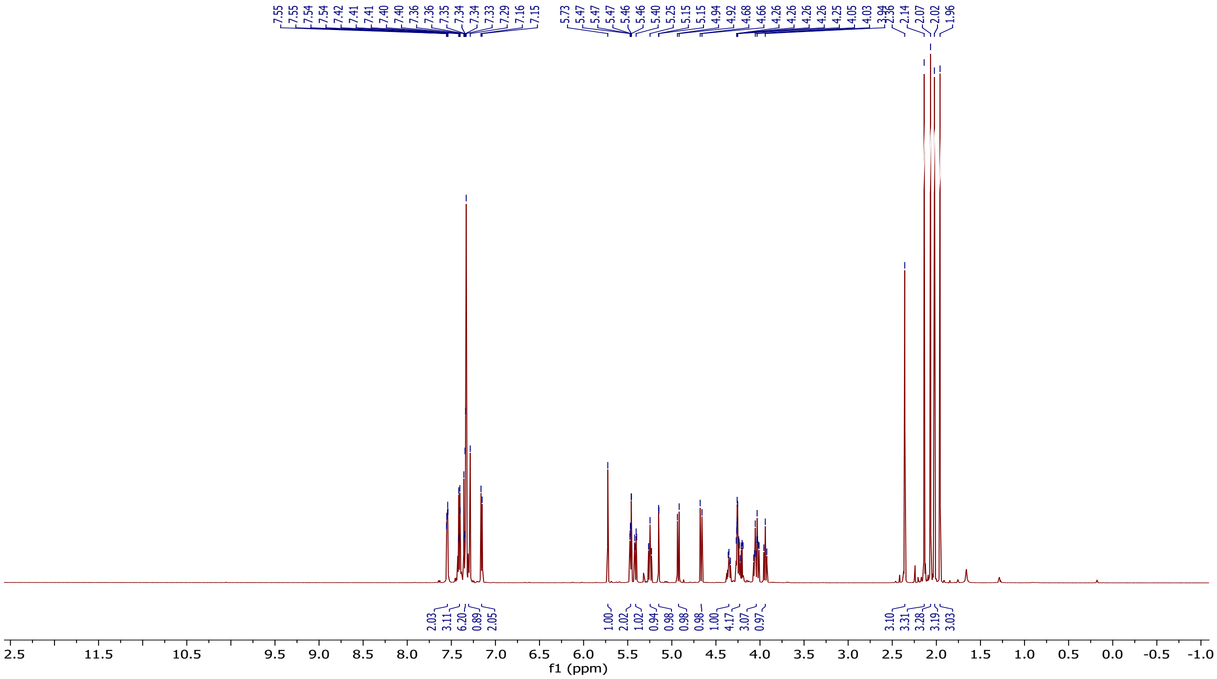

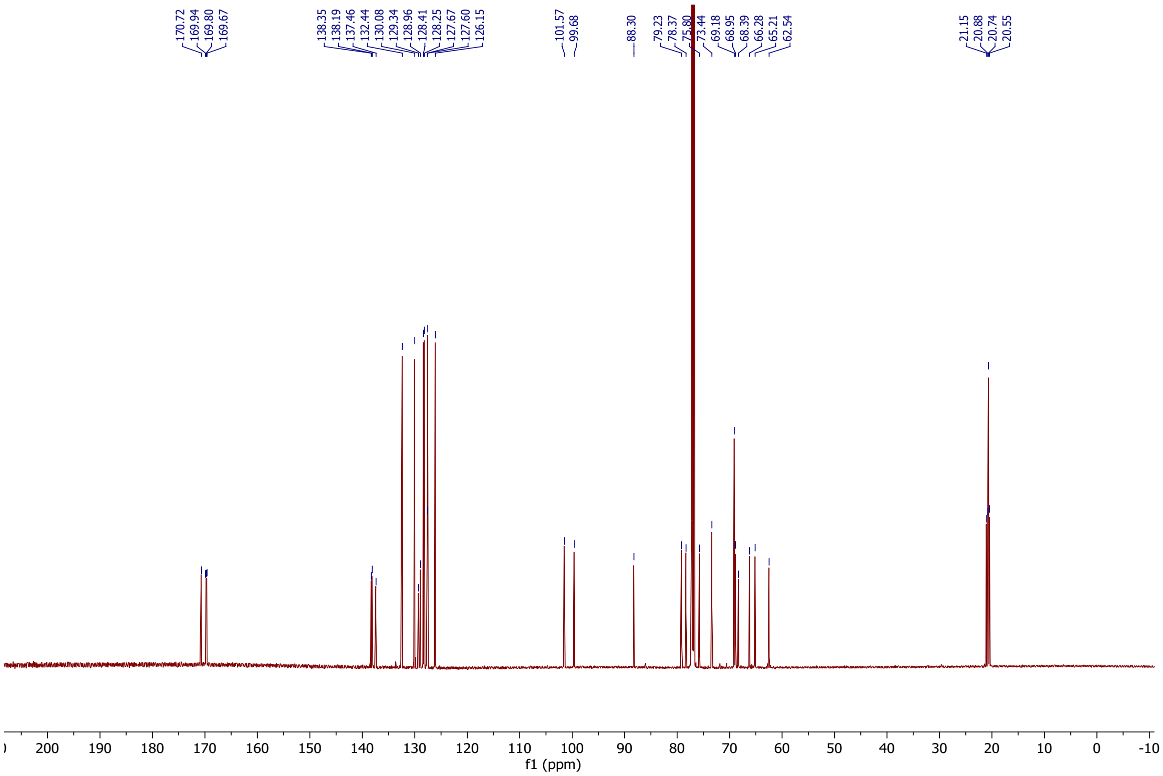

Figure S9: ^{1}H and ^{13}C NMR of Compound **3** ($CDCl_3$, 600 MHz)

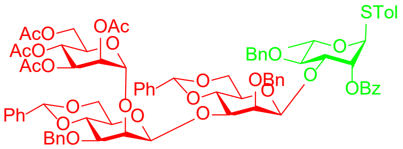

3

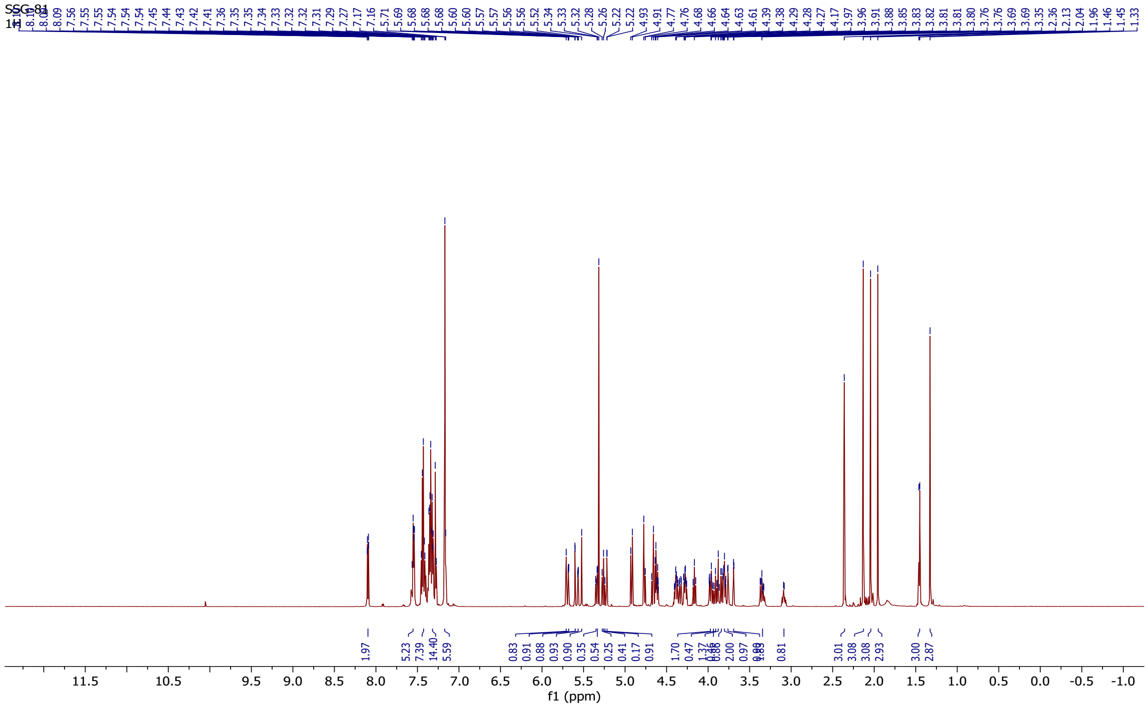

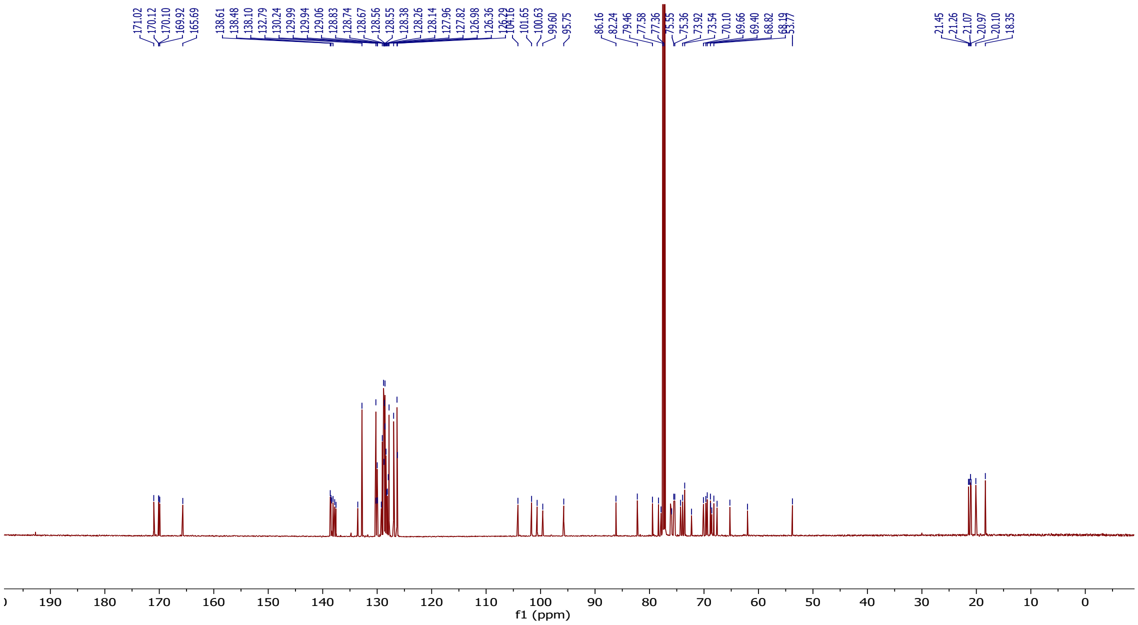

Figure S10: ^{1}H and ^{13}C NMR of compound **2** ($CDCl_3$, 600 MHz)

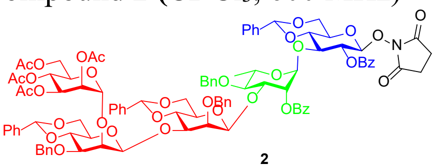

2

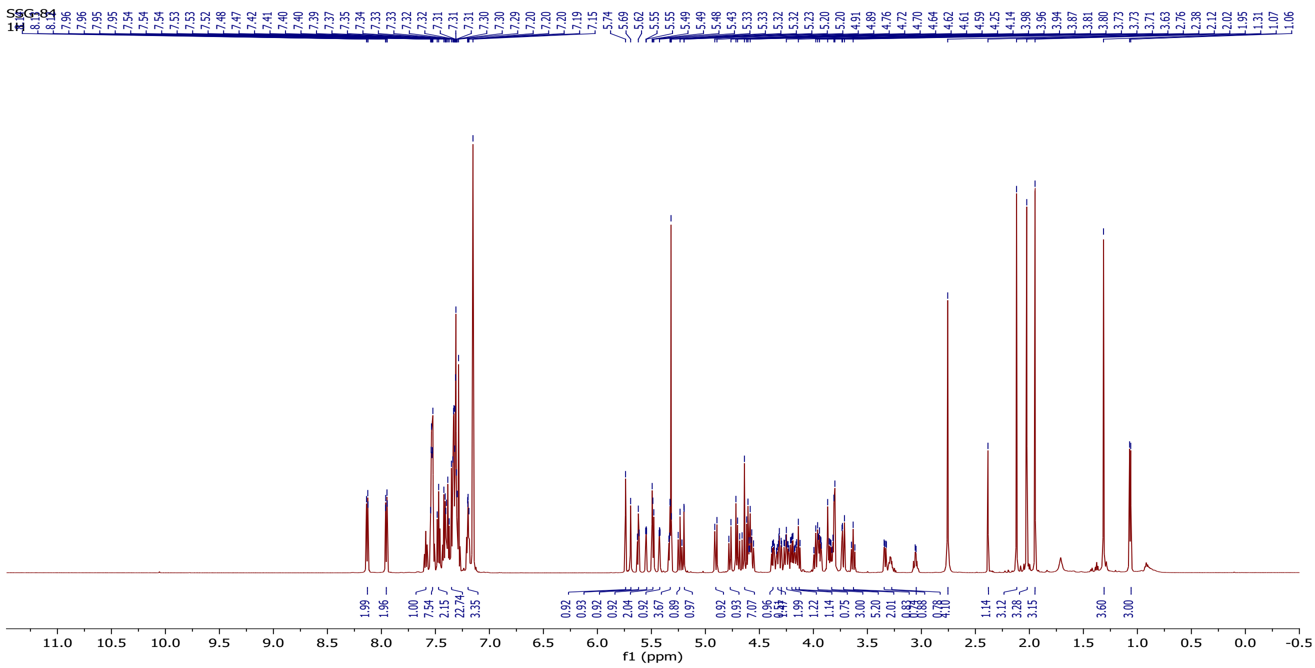

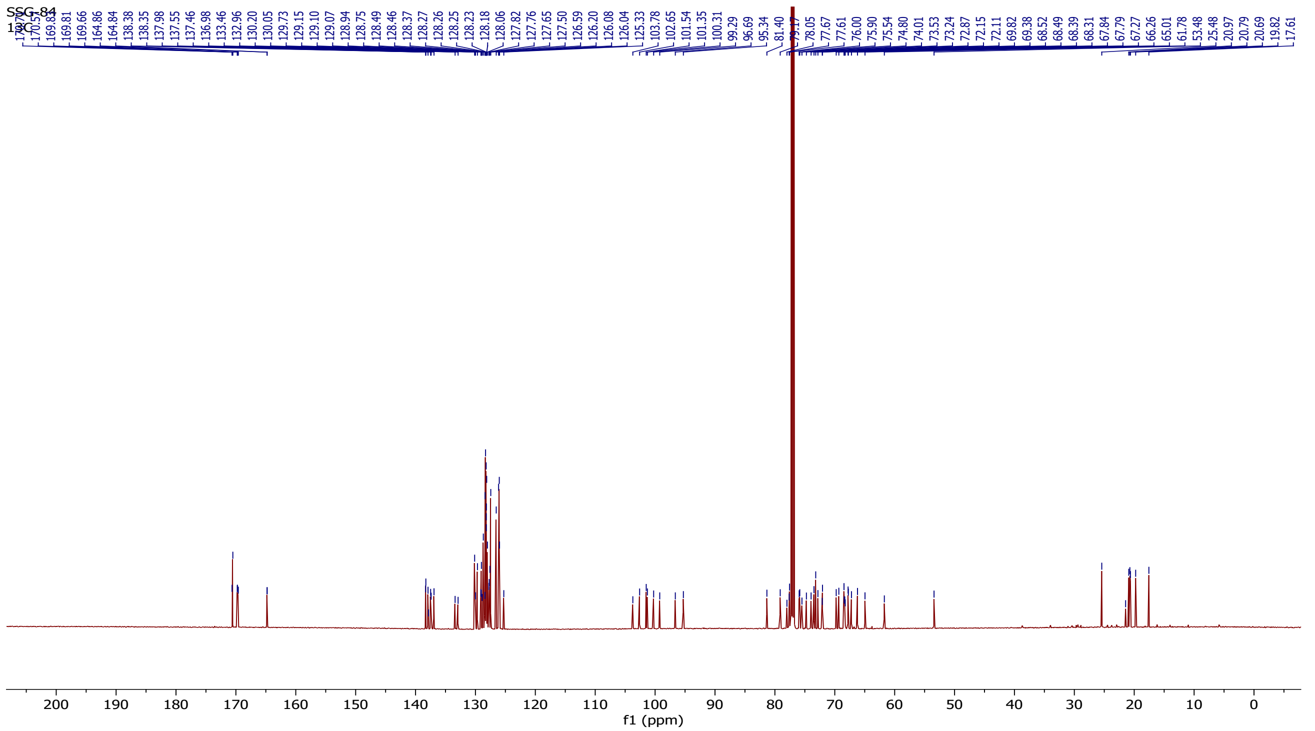

Figure S11: ^{1}H and ^{13}C NMR of compound **1** (D_2O, 600 MHz)

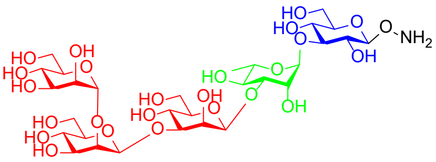

1

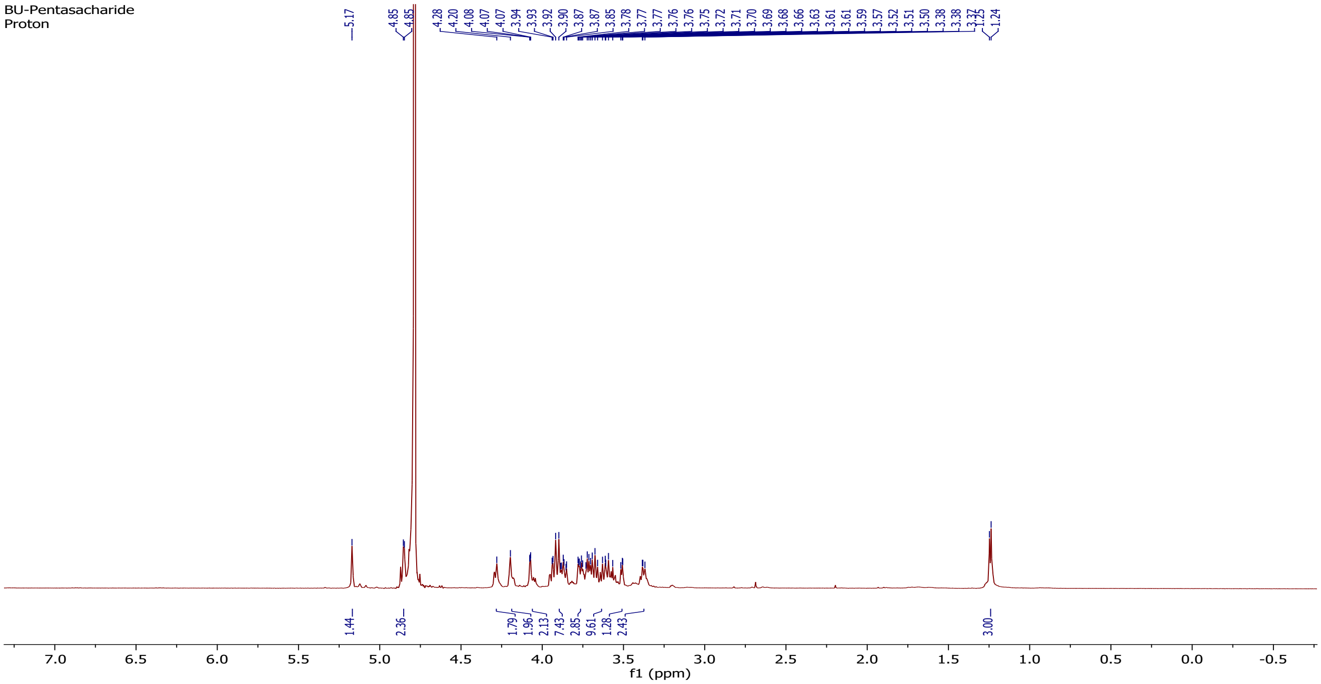

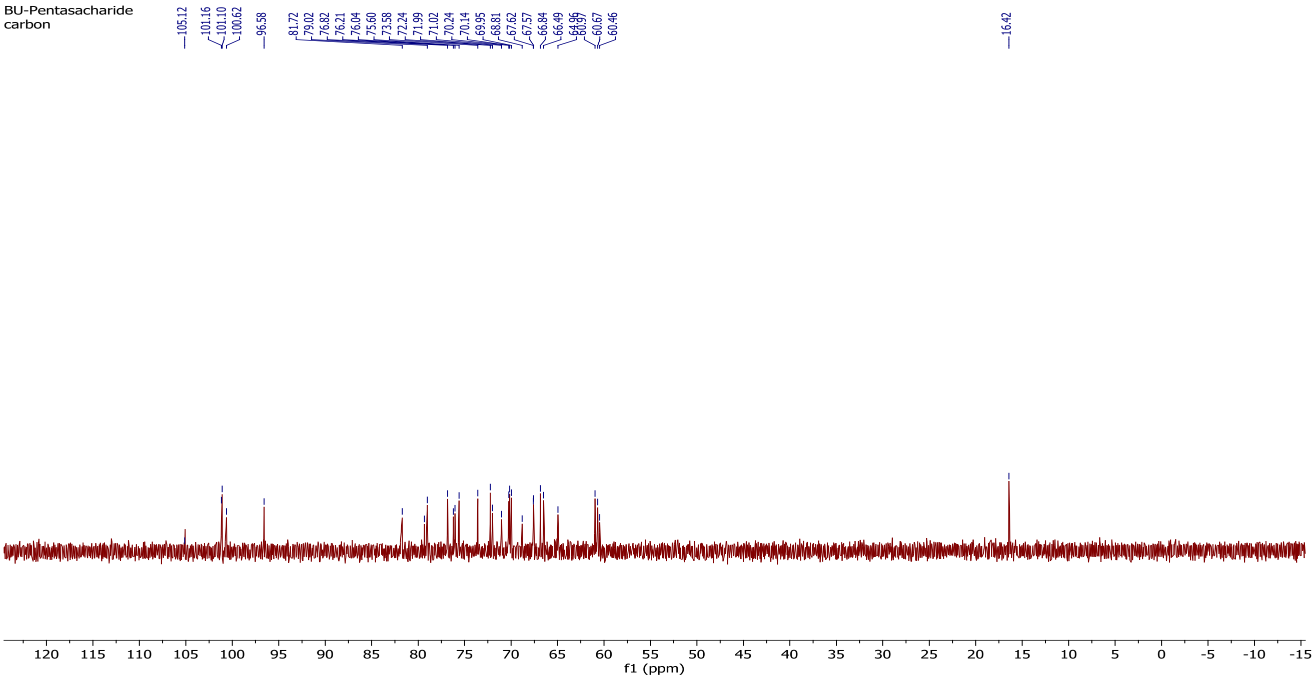

Figure S12: HRMS Data of Compound **1**

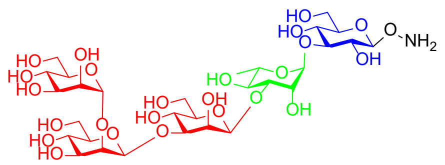

1

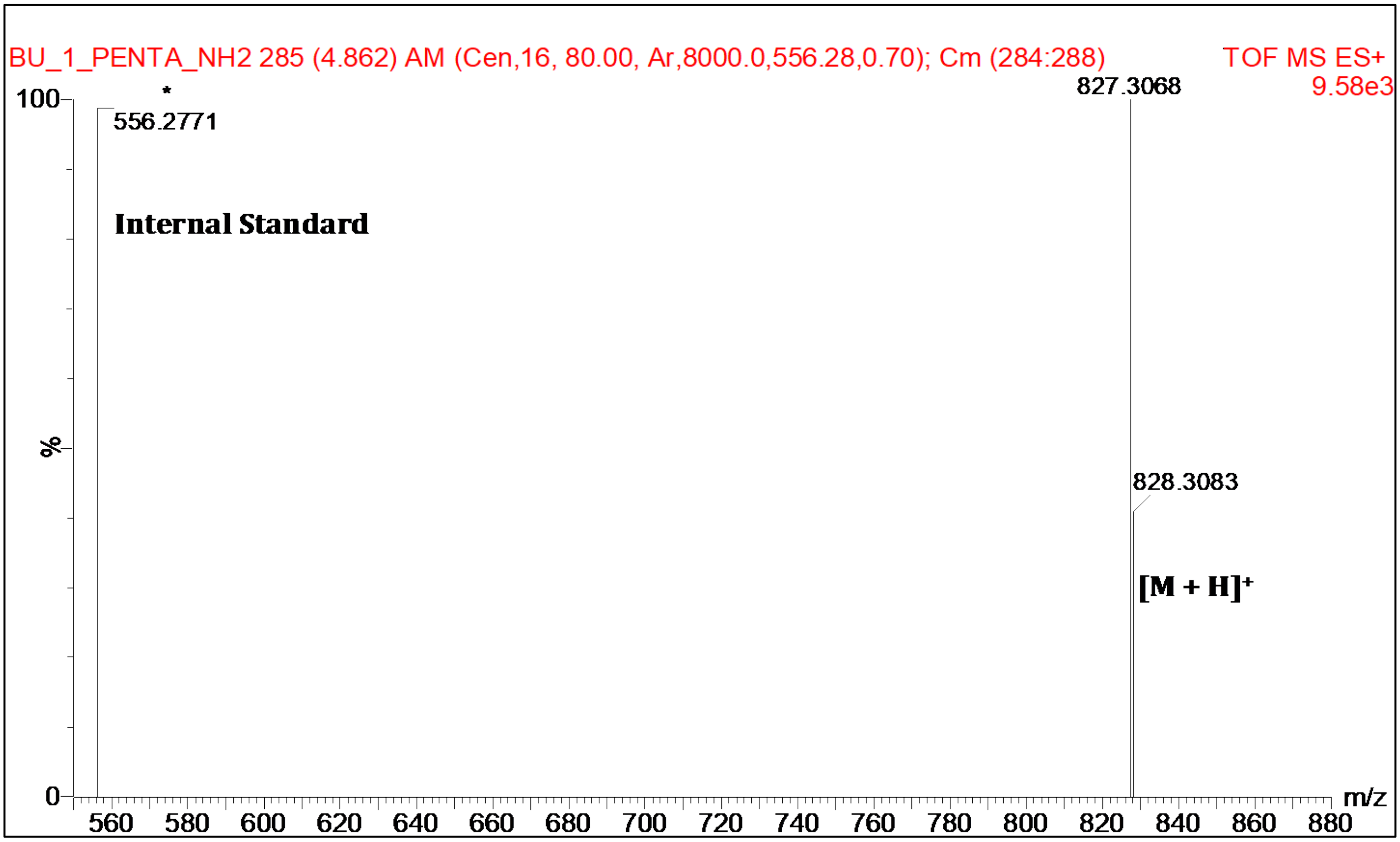

BU_1_PENTA_NH2 285 (4.862) AM (Cen,16, 80.00, Ar,8000.0,556.28,0.70); Cm (284:288)
TOF MS ES+
9.58e3
100
*
556.2771
827.3068
Internal Standard
%
828.3083
[M + H]+
0
560
580
600
620
640
660
680
700
720
740
760
780
800
820
840
860
880
m/z

Figure S13: ESI-MS of Compound **15**

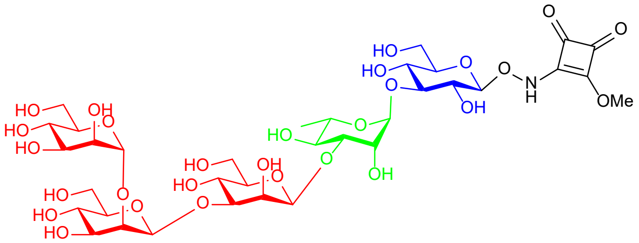

15

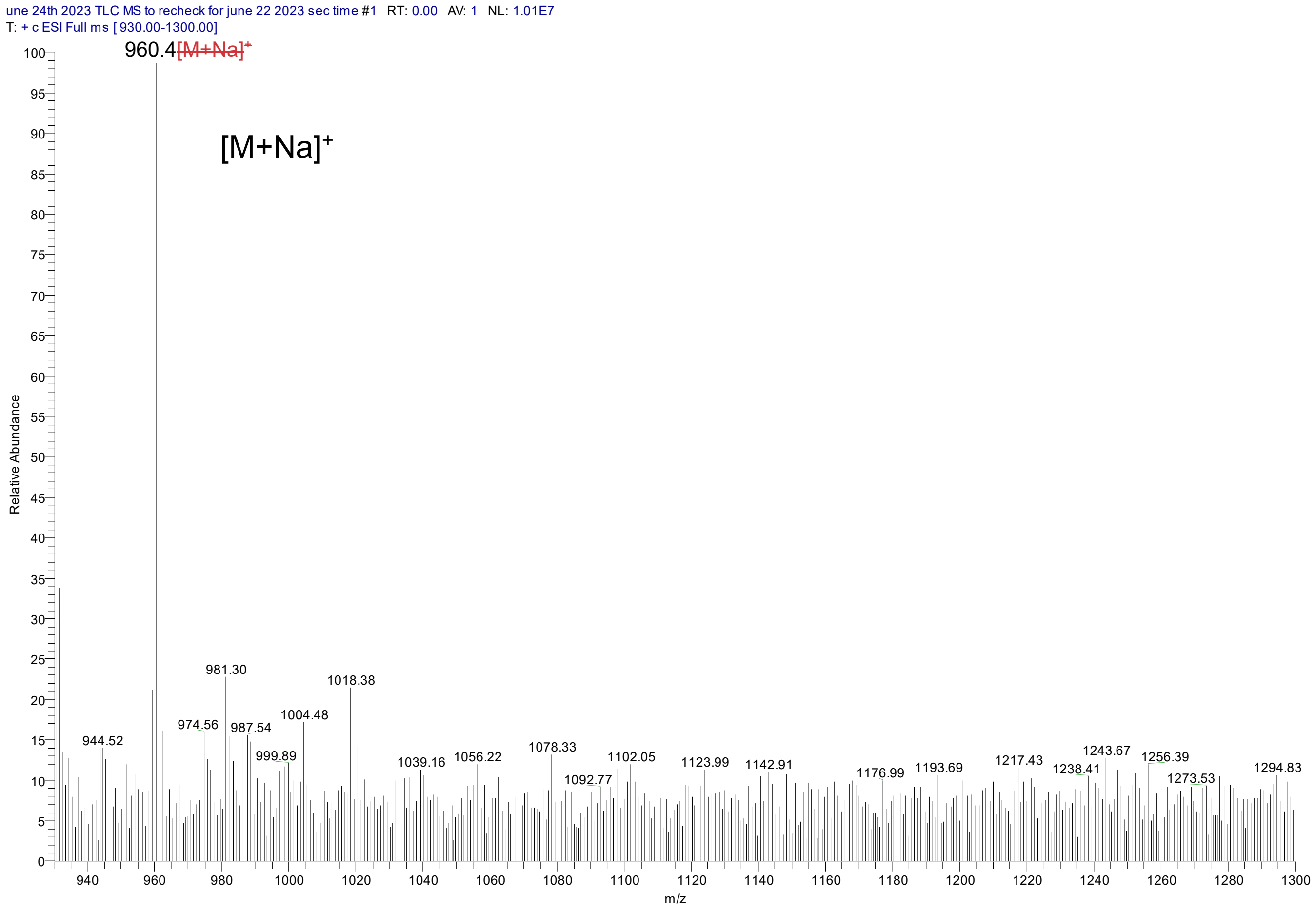

Figure S14: ESI-MS of Psl-Biotin conjugate **18**

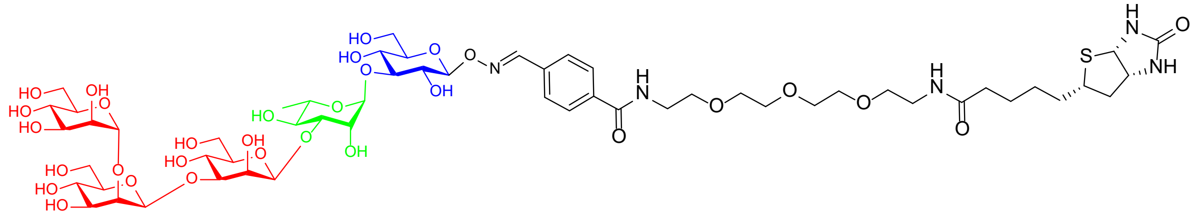

18

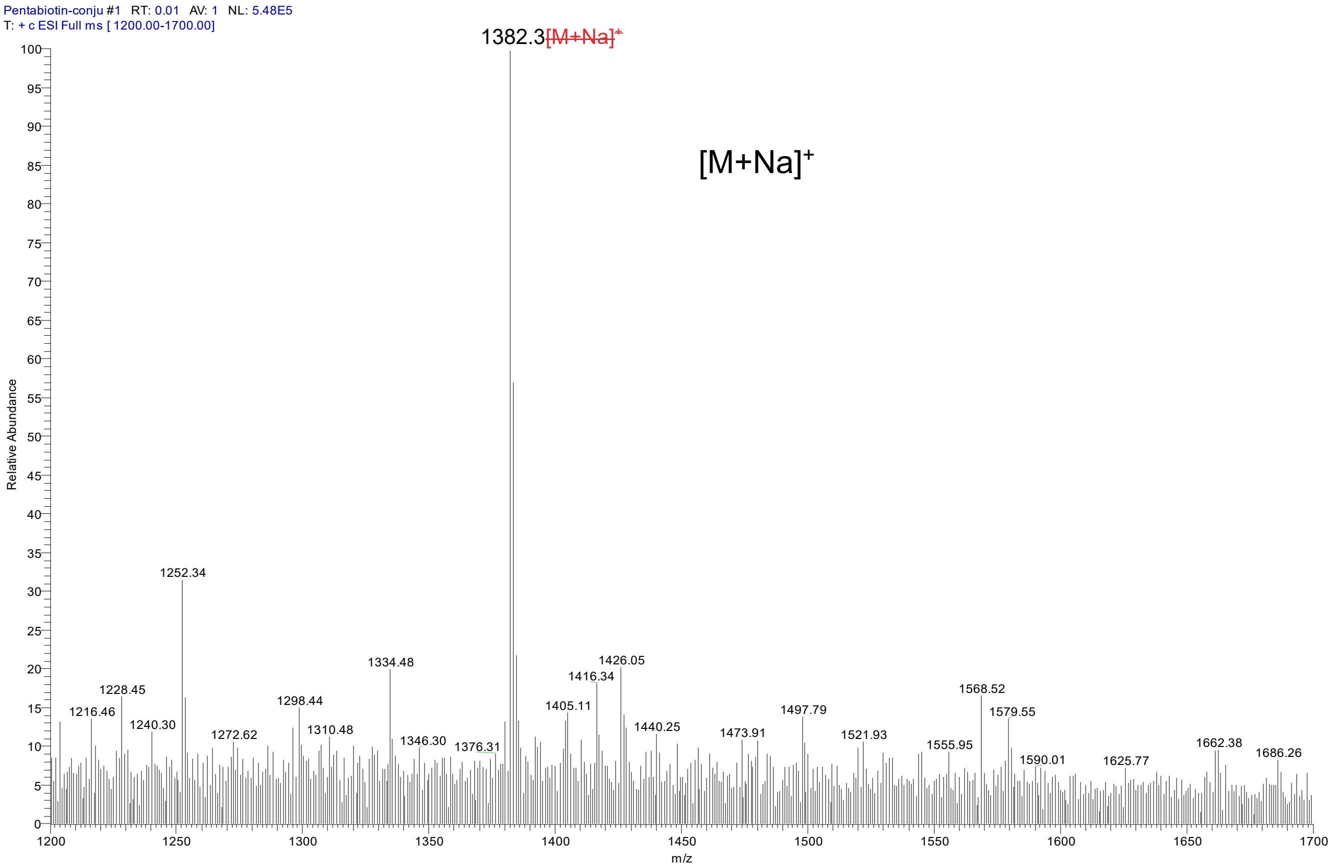

Figure S15: SDS PAGE of Compound **16**

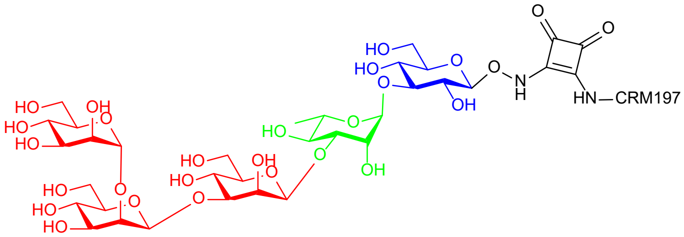

16

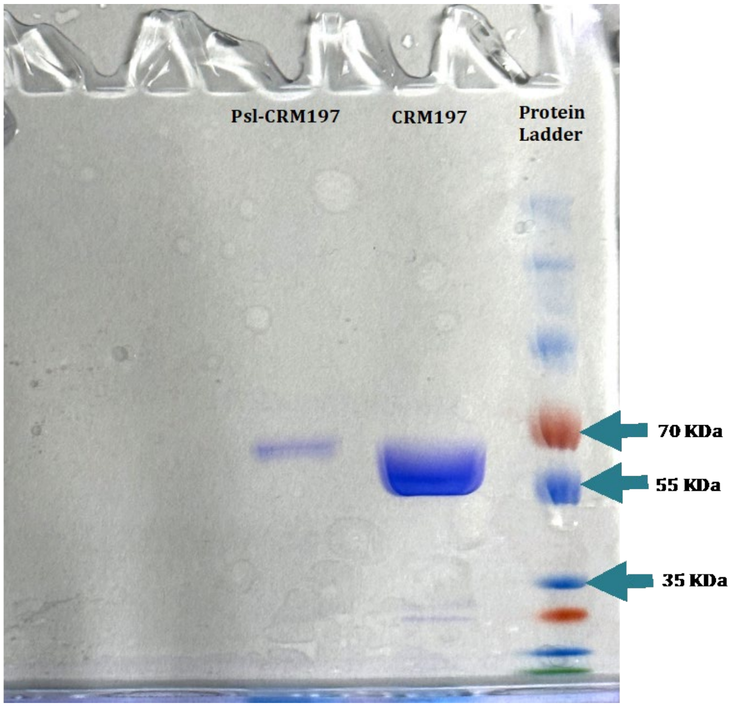

Figure S16: MALDI-TOF data of Compound **16**

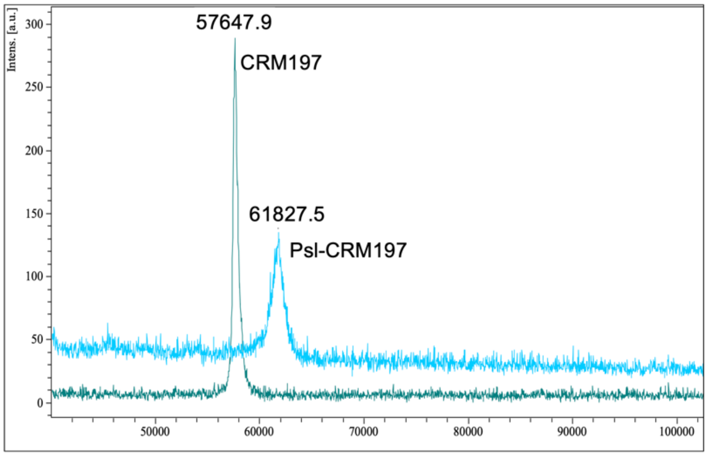

Figure S17: SDS-PAGE of BSA conjugate to Psl (Compound **17**)

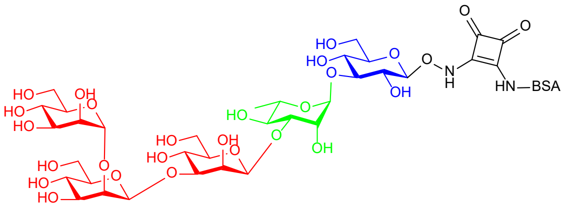

17

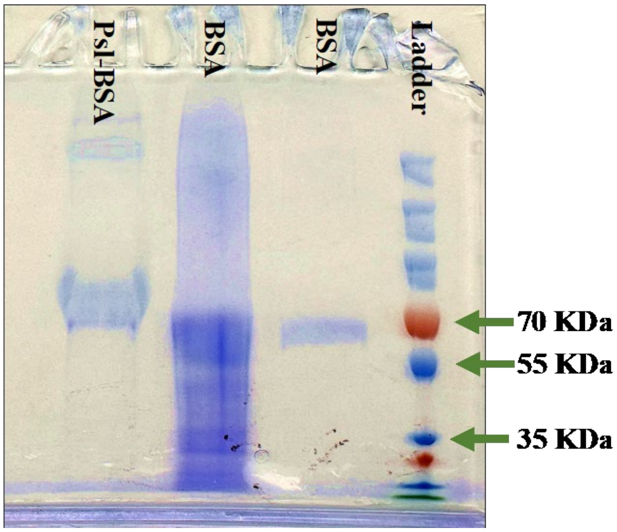

Figure S18: MALDI-TOF of BSA Conjugate to Psl (Compound **17**)

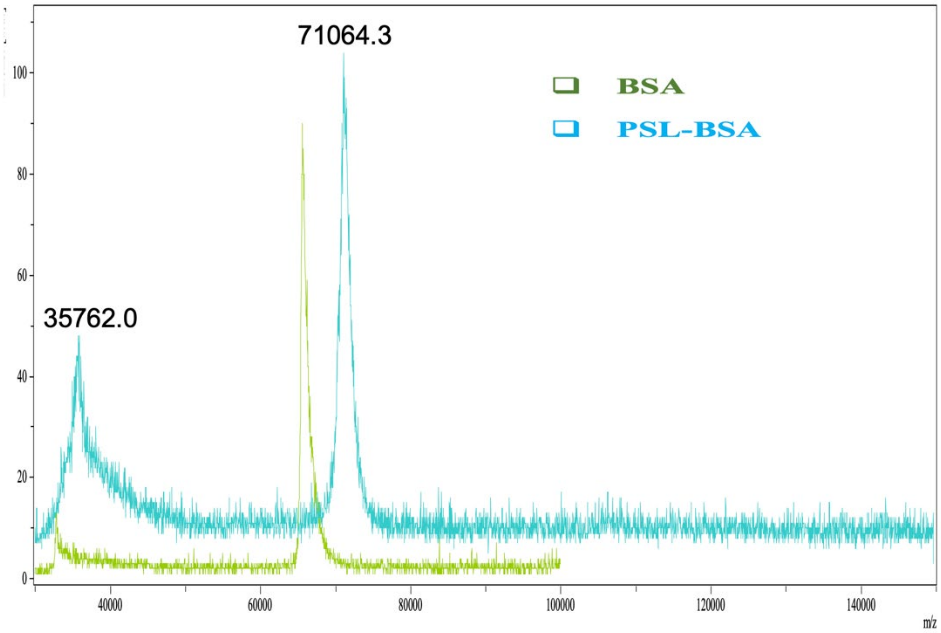

Table S1: DLS data for QS-21 and QS-21 + Psl-CRM197 (**16**) liposomes.

Liposome formulation	R_h (nm)	ζ (V)
QS-21 liposome (Control)	500.88	0.0057 ±0.0014
QS-21+Psl-CRM197 (**16**) Liposome	1029.51	0.0016 ±0.0014

Figure S19A: Vaccine DLS Data (DMPG/DMPC/Cholesterol/Pam3CysSK4/QS-21/**16**): Phase Plot

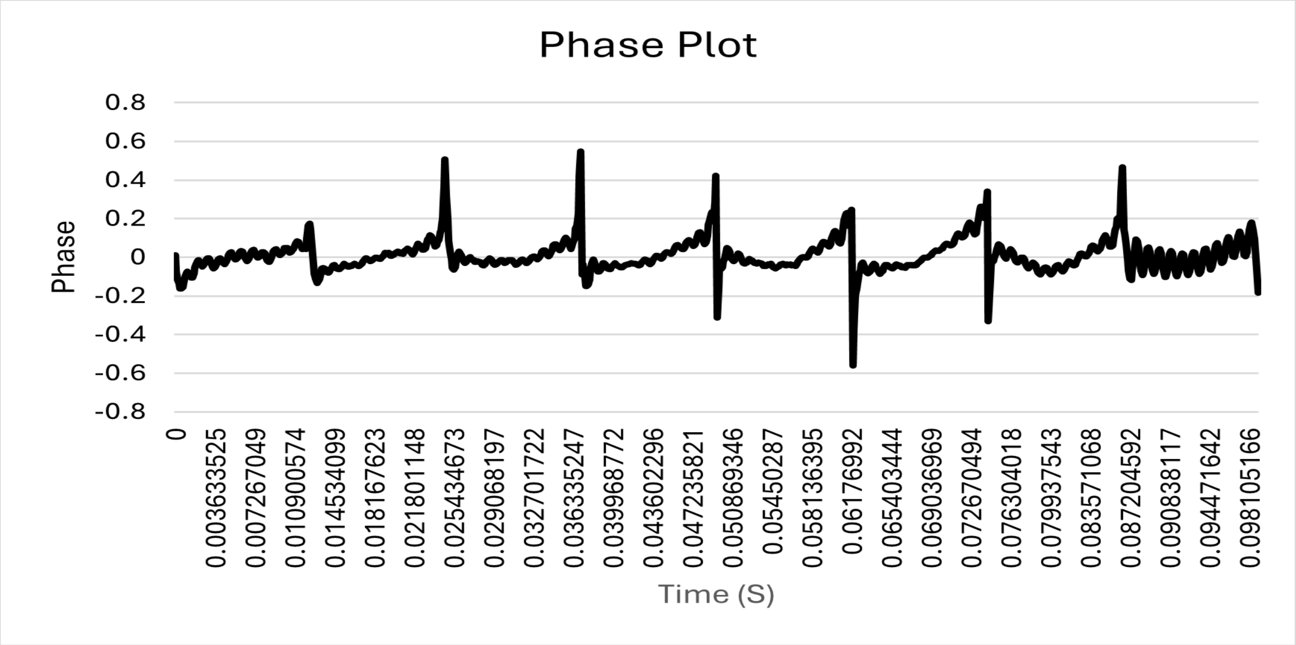

Figure S19B: Vaccine DLS Data (DMPG/DMPC/Cholesterol/Pam3CysSK4/QS-21/**16**): Size Distribution

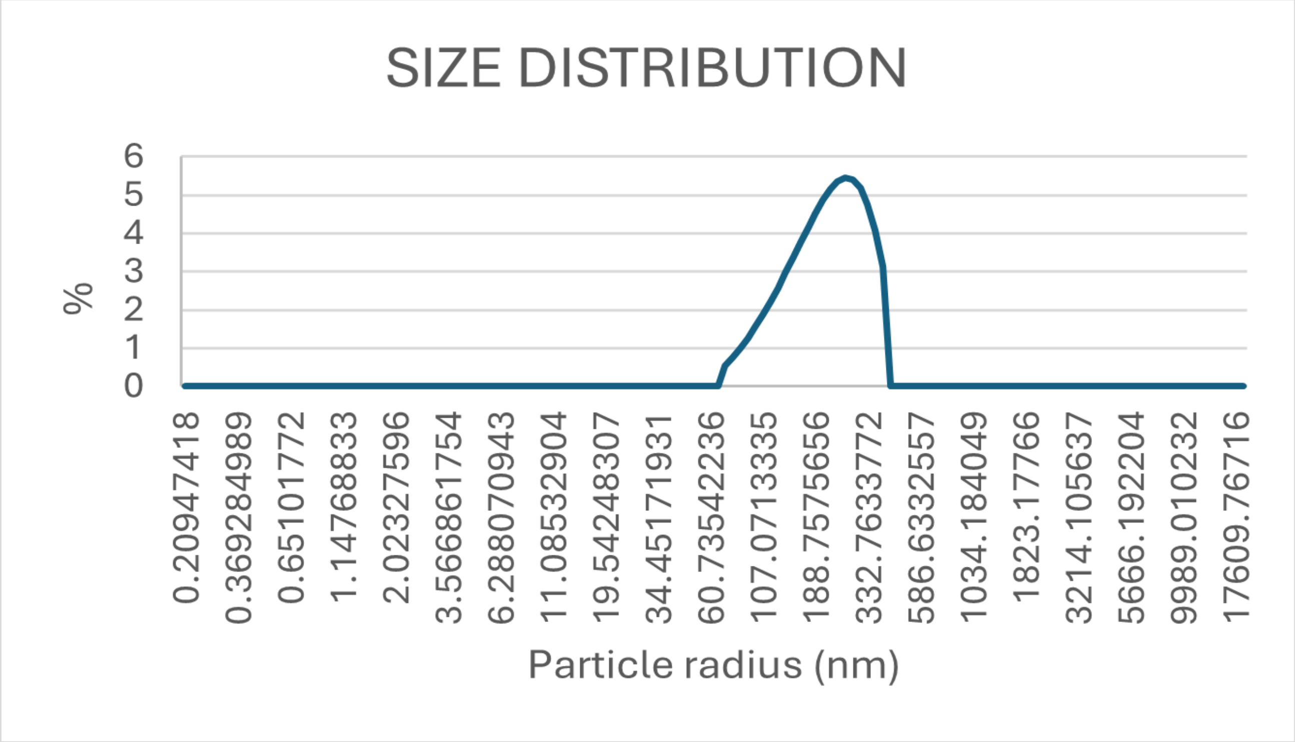

Figure S19C: Vaccine DLS Data (DMPG/DMPC/Cholesterol/Pam3CysSK4/QS-21/**16**): Zeta Potential

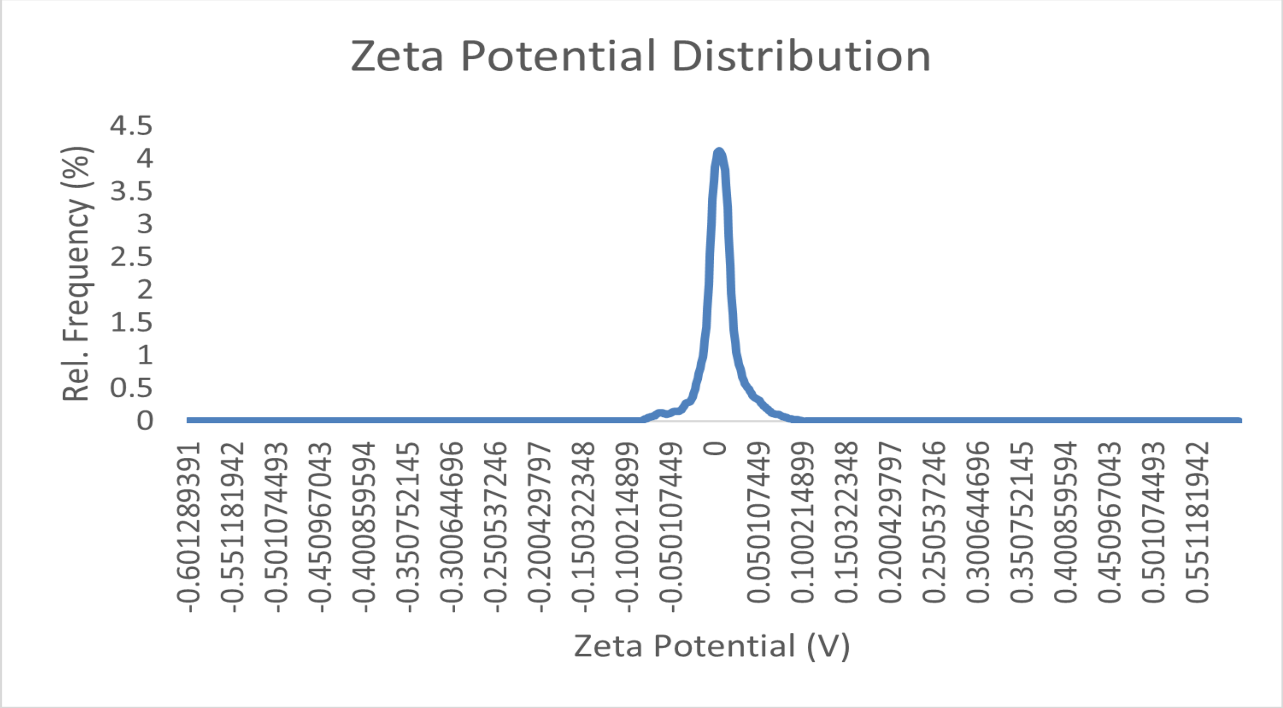

Figure S19D: Vaccine DLS Data (DMPG/DMPC/Cholesterol/Pam3CysSK4/QS-21/**16**): Zeta Potential

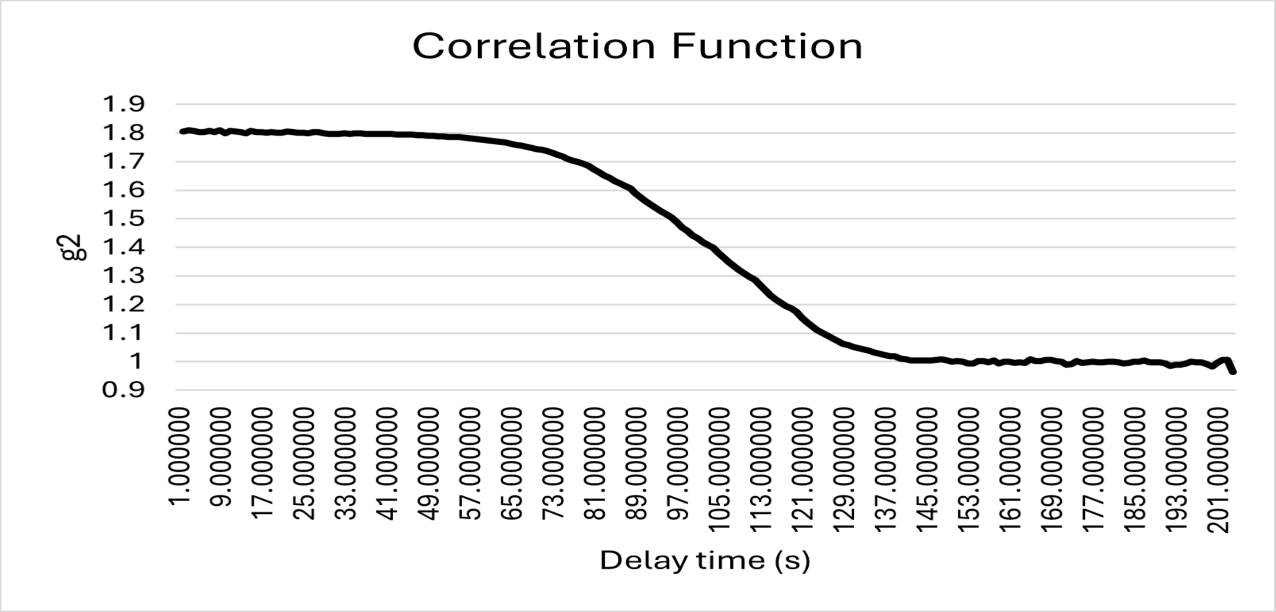

Supplement: Supplementary file 1 [file molecules-30-01720-s001.zip › molecules-3520562-supplementary.pdf]
